# Valorization of *Zanthoxylum bungeanum* Maxim. Leaf By-Products: Comparative Aroma Profiling with Pericarps Across Extraction Strategies

**DOI:** 10.3390/foods15122243

**Published:** 2026-06-22

**Authors:** Zongyuan Wu, Chenxi He, Yunlong Xiao, Yinhao Xue, Rongrong Zhang, Shouan Ming, Yanxia Cong, Weinong Zhang

**Affiliations:** 1 College of Food Science and Engineering, Wuhan Polytechnic University, Key Laboratory for Deep Processing of Major Grain and Oil, Ministry of Education, Hubei Key Laboratory for Processing and Transformation of Agricultural Products, Wuhan 430048, China; wzytotti@whpu.edu.cn (Z.W.); hechenxi0817@163.com (C.H.); xueyinhao04@163.com (Y.X.); rongrzh@163.com (R.Z.); 2 Shenzhen Boton Flavors & Fragrances Co., Ltd., Shenzhen 518055, China; xiaoyl0913@163.com; 3 Hubei Mingchuang Agricultural Technology Development, Co., Ltd., Huanggang 438400, China; mingshouan@163.com

**Keywords:** *Zanthoxylum bungeanum* Maxim., leaf by-products, resource valorization, odor activity value, aroma recombination

## Abstract

While *Zanthoxylum bungeanum* Maxim. (*Z. bungeanum*) pericarps are a globally prized spice, their leaves are frequently discarded as agricultural waste. This study systematically characterizes the aromatic potential of leaf by-products compared with traditional pericarps under diverse extraction strategies, utilizing an integrated flavoromics and sensomics approach. Qualitative GC-MS-O analysis revealed that leaf-derived fractions possess superior aromatic diversity: leaf essential oil and volatile solvent extract yielded 71 and 68 odorants, respectively, significantly surpassing pericarp counterparts (65 and 43 compounds). Concurrently, HS-GC-IMS profiling confirmed that targeted extraction allows leaf-derived flavors to replicate and exceed traditional spice complexity. Specifically, the leaf solvent extract achieved aromatic parity with pericarps by effectively mirroring the core spicy–citrus profile through cuminaldehyde and limonene retention. Conversely, distilled leaf essential oil unlocked a distinctive herbal–woody sensory innovation, driven by eucalyptol and a broader variety of aldehydes and ketones. Sensomics validation, incorporating aroma recombination, omission experiments, and partial least-squares regression modeling, conclusively identified β-myrcene, limonene, caryophyllene, and humulene as core molecular markers dictating these perceptual shifts. Ultimately, this research provides a robust theoretical foundation for upcycling *Z. bungeanum* leaves into valuable flavoring resources, facilitating circular bio-economy practices by delivering functional equivalence and entirely novel sensory experiences for the global food industry.

## 1. Introduction

*Zanthoxylum bungeanum* Maxim. (*Z. bungeanum*), a core spice in Asian culinary traditions, is globally prized for its signature tingling sensation and complex aromatic profile [1]. These sensory attributes are primarily governed by its volatile fractions, which are synthesized across various plant tissues, including roots, stems, leaves, and fruits [2]. In the food industry, these components are essential not only for their aromatic contribution to seasoning oils and functional foods but also for their potent antioxidant and antimicrobial properties, which serve as natural alternatives to synthetic preservatives [3]. However, while fruit pericarps are high-value commercial commodities, the leaves of *Z. bungeanum*, despite being an abundant agricultural by-product, are frequently discarded as waste, leading to significant resource underutilization and environmental pressure [4].

The chemical fingerprint of *Z. bungeanum* volatiles traditionally encompasses a diverse array of terpenoids, alcohols, aldehydes, and esters [5]. To harness these compounds, classical techniques such as steam distillation (SD) and solvent extraction (SE) remain the industrial mainstays due to their operational simplicity and cost-effectiveness [6]. According to international standards (e.g., ISO 9235) [7], it is critical to distinguish between the resulting products: distillation yields essential oils (EOs), whereas solvent extraction produces volatile extracts (VEs) or oleoresins [8]. Crucially, the choice of extraction strategy acts as a molecular tailoring tool, significantly dictating the recovery of specific aroma-active compounds [9]. For instance, solvent extraction may better retain high-boiling-point markers, while distillation is favored for isolating high-purity volatile terpenoids. Understanding how diverse extraction strategies can reshape the molecular flavor blueprint of these overlooked leaf by-products is vital for their successful valorization. By strategically selecting extraction methodologies, it is possible to bridge the sensory gap between agricultural by-products and premium commercial extracts.

Recent advances in flavoromics have integrated multiple analytical platforms to decode complex food matrices [10,11]. Modern aroma profiling relies on a systematic workflow that couples versatile sample preparation methods (e.g., HS, HS-SPME, and SBSE, occasionally with derivatization) [12] with gas chromatography (GC) separation and advanced detection systems like MS or IMS. Techniques such as gas chromatography–mass spectrometry (GC-MS), electronic nose (E-nose), and headspace gas chromatography–ion mobility spectrometry (HS-GC-IMS) have been employed to fingerprint *Zanthoxylum* species [13]. While these methods provide robust chemical profiling, they are inherently limited in their ability to translate instrumental data into actual human sensory perception [14,15]. To bridge this gap, gas chromatography–mass spectrometry–olfactometry (GC-MS-O) combined with the sensomics approach, including aroma extract dilution analysis (AEDA) and odor activity value (OAV) calculations, is essential for identifying the key odorants that truly define a food’s sensory identity [16,17]. Despite extensive research on pericarps, the molecular basis of the flavor potential in *Z. bungeanum* leaves remains poorly understood. Specifically, it remains unclear whether leaf extracts can achieve aromatic parity with pericarps or if they offer entirely novel sensory experiences. Investigating these leaf by-products as a sustainable source of food flavorings aligns with the urgent global demand for circular bio-economy practices in the food sector.

Therefore, the present study aims to establish a comprehensive flavoromics and sensomics framework to systematically decode the valorization potential of *Z. bungeanum* leaf by-products. The novelty of this research is anchored in a direct matrix-matched comparison between discarded leaves and commercial pericarps, coupled with the demonstration of how divergent extraction methods act as “molecular tailoring” tools. Furthermore, this study integrates advanced sensomics—specifically GC-MS-O, OAV, and aroma recombination/omission—to scientifically validate these perceptual differences. By conducting a matrix-matched comparison between the volatile fractions (EOs and VEs) of leaves and those of the commercially established pericarps, this study employed: (i) sensory profiling and E-nose analysis to establish the overall aroma landscape of the four distinct matrice; (ii) HS-GC-IMS for rapid volatile fingerprinting and pattern recognition; (iii) GC-MS-O (AEDA) to identify and quantify the key odorants within each extract; and (iv) recombination and omission experiments to validate the contribution of individual molecular markers to the characteristic *Z. bungeanum* aroma. Our objective was to demonstrate that through strategic extraction-driven tailoring, leaf by-products can be transformed into high-value flavoring resources that transcend their status as agricultural waste. Ultimately, we hypothesize that leaf-derived fractions possess the molecular flexibility to either effectively replicate the signature spicy–citrus notes of traditional pericarps or unlock distinctive herbal–woody profiles that are entirely absent in pericarp-based products. This research provides a robust theoretical framework for transforming agricultural waste into versatile, valuable flavor resources for the global food and seasoning industries.

## 2. Materials and Methods

### 2.1. Materials

Fresh leaves and pericarps of *Z. bungeanum* (Dwarf thornless “Dahongpao” Chinese prickly ash) were obtained from Hubei Mingchuang Agricultural Technology Development Co., Ltd. (Huanggang, China). More details were provided in the Supplementary Material. Authentic standards were used to positively confirm the identified odor compounds. α-Pinene (purity 98.3%), β-myrcene (90.5%), limonene (94.8%), linalool (97.5%), terpinen-4-ol (99.9%), carvone (96.1%), linalyl acetate (98.8%), α-terpinyl acetate (94.3%), caryophyllene (98.5%), humulene (99.1%), styrene (99.7%), o-cymene (98.7%), γ-terpinene (97.7%), terpinolene (97.0%), cuminaldehyde (94.9%), piperitone (95.6%), neryl acetate (98.9%), geranyl acetate (99.0%), caryophyllene oxide (98.0%), methyl palmitate (99.7%), dibutyl phthalate (99.1%), 2-methyl-3-heptanone (99.0%) and *N*-alkanes (C7-C40) were purchased from Shanghai ANPEL Laboratory Technologies Inc. (Shanghai, China). Petroleum ether (purity 98%) were obtained from Tianjin Kemiou Chemical Reagent Co, Ltd. (Tianjin, China). N-ketones (C4-C9) was purchased from Shandong Haineng Scientific Instrument Co., Ltd. (Dezhou, China).

### 2.2. Preparation of Volatile Fractions

The pericarps and leaves of *Z. bungeanum* were obtained from Hubei Mingchuang Agricultural Technology Development Co., Ltd. (Huanggang, China). To ensure extraction efficiency, fresh leaves and dried pericarps (60 °C) were used immediately or stored at −20 °C. Following the aforementioned extraction methods [6], four types of volatile samples were prepared: solvent-extracted pericarp VEs (S1), steam-distilled pericarp EOs (S2), steam-distilled leaf EOs (S3), and solvent-extracted leaf VEs (S4). For the preparation of VEs (S1 and S4), 15 g of fresh leaves or dried pericarps were submerged in petroleum ether (105 mL for leaves; 180 mL for pericarps) within a 500 mL flask. The mixture underwent ultrasonic-assisted extraction for 30 min, followed by refluxing in a water bath at 40 °C for 2 h (timed from the onset of boiling). The resulting solution was filtered, and the filtrate was dehydrated with 5 g of anhydrous sodium sulfate at −20 °C overnight. After a secondary filtration, the solvent was removed via rotary evaporation under reduced pressure at 40 °C to yield the corresponding VEs (S1 and S4). For steam distillation (S2 and S3), 150 g of fresh leaves or 15 g of pericarps were placed in a specialized steam bulb. Distilled water (900 mL for leaves; 210 mL for pericarps) in the boiling flask was heated to generate continuous steam, which passed through the leaf matrix for 8 h. The EOs (S2 and S3) were subsequently recovered from the oil-water separator.

### 2.3. Sensory Evaluation

The sensory evaluation was performed referring to prior sensory reports with slight modification [18]. Aroma profiling analysis (APA) was performed by 15 trained assessors (7 males and 8 females, 22–32 years old) in a sensory evaluation room at 24 ± 1 °C, during which odor attributes were rated on an intensity scale of 1 unit from 0 (not perceivable) to 5 (strongly perceivable). Odor attributes and corresponding standards used in the training were shown in Table S1. All evaluators received and passed perception training prior to participating in the experiment. Samples (10 μL) were placed into 15 mL glass vial, respectively, and then evaluated for overall odor characteristics according to APA. Detailed description of the sensory evaluation was provided in the Supplementary Material.

### 2.4. Electronic Nose (E-Nose) Analysis

The volatile fingerprints of S1–S4 were distinguished using a cNose-18 system (Shanghai Baosheng Industrial Development Co., Ltd., Shanghai, China) equipped with 28 metal oxide sensors. Following the method of Jia et al. [19], 1.0 μL of the sample was placed in a 10 mL headspace vial. The maximum response values of each sensor were recorded during the analysis, and each sample was tested in triplicate. A detailed description of the E-nose analysis was provided in the Supplementary Material.

### 2.5. HS-GC-IMS Analysis

Volatile profiling was performed using an HS-GC-IMS system (FlavourSpec^®^, G.A.S., Dortmund, Germany) according to Wu et al. [20] with modifications. Samples (0.1 μL) were incubated in a headspace vial at 35 °C for 10 min (500 rpm). The temperature and volume of the headspace injection were 40 °C and 100 µL, respectively. Ultrapure nitrogen (purity ≥ 99.999%) was used as a carrier gas and the initial flow rate set to 1 mL/min, raised to 2 mL/min within 3 min, then raised to 10 mL/min within 5 min, then raised to 12 mL/min within 7 min, then raised to 100 mL/min within 20 min, and finally raised to 150 mL/min within 30 min. The drift gas flow rate was established at 150 mL/min. The MXT-5 column (15 m × 0.53 mm, 1 µm, Restek Corporation, Bellefonte, PA, USA) and IMS temperature were kept at 60 °C and 45 °C. The data were qualitatively analyzed using VOCal software (version 0.4.03), which is equipped with NIST 11 and IMS databases. Detailed description of the HS-GC-IMS conditions was provided in the Supplementary Material.

### 2.6. Isolation of Aroma Compounds by SPME

For GC-MS-O analysis, volatiles were extracted using headspace solid-phase microextraction (HS-SPME) [21]. Samples (1 μL) were sealed in 20 mL vials and equilibrated. A 50/30 μm CAR/DVB/PDMS fiber (Supelco, Inc., Bellefonte, PA, USA) was exposed to the headspace at 80 °C for 20 min and subsequently desorbed in the GC injector at 270 °C for 10 min. Three parallel tests were performed for each sample. More details were provided in the Supplementary Material.

### 2.7. Identification of the Odor Compounds by GC-MS-O

Evaluation of highly volatile odor compounds was accomplished by means of Agilent 7890A-5975C GC-MS (Agilent Technologies Inc., Santa Clara, CA, USA) in combination with an olfactometer (ODP3, Gerstel, Mülheim an der Ruhr, Germany) [22]. The SPME extract was injected into HP-5 (30 m × 250 µm × 0.25 µm). The GC analysis was performed as follows: split ratio, 75:1; injection temperature, 270 °C; oven temperature program, 40 °C and hold for 1 min, up to 170 °C at 3 °C/min, up to 270 °C at 8 °C/min and hold for 5 min; the flow rate of carrier gas (high-purity helium), 1.3 mL/min. MS conditions: ion source temperature, 250 °C; quadrupole temperature, 150 °C; EI energy, 70 eV; scan range, *m*/*z* 35–550; solvent delay time, 3.0 min. Odor compounds with odor characteristics were recorded by at least three experienced team members (two females and one male, 24–32 years old). The data were qualitatively analyzed using MSD Chemstation (version F.01, Agilent, USA), which is equipped with NIST11.L databases. Detailed description of the GC-MS-O conditions was provided in the Supplementary Material.

### 2.8. Aroma Extract Dilution Analysis (AEDA)

The flavor dilution (FD) factors of aroma compounds were determined by AEDA GC-MS-O referring to previous method [23]. For dilution through adjusting GC injector split ratio, the volumes of the EOs were 0.1 μL, and the GC injector split ratio was set as 3:1, 9:1, 27:1, 81:1, 243:1, 729:1, and 2187:1. In the olfactometry detection port, the 3 panelists sniffed each solution from the lowest to highest dilutions until no odor was perceived. The flavor dilution (FD) factor was obtained for each aroma compound as the last dilution when it was detectable.

### 2.9. Quantitative Method

Calibration standard curves were carried out by plotting the area ratio of external standard to internal standard (IS) against their corresponding concentration ratio. Briefly, 1 μL external standards (21 standards in Table S2) with different concentration levels and 10 μL 2-methyl-3-heptanone (0.816 mg/mL) were added to the vials with glass microintubation tubes. The mixtures were analyzed by GC-MS-O on HP-5 column using selected ion monitoring (SIM) mode [19]. Then, the response factor (Rf) was calculated, as provided in Table S2. Additionally, the IS method (2-methyl-3-heptanone as an IS) was employed for the quantification of the remaining compounds. The concentration was calculated according to the previous method [21]. The recovery (%) of the odor compound was determined according to the previous method [19]. The experiment was replicated in triplicate.

### 2.10. Analysis of Odor Activity Values (OAVs)

Odor thresholds (OTs) were determined in a refined, odorless camellia oil matrix using the 3-alternative forced choice (3-AFC) method [24]. OT determinations were conducted in a controlled sensory evaluation room at 25 ± 1 °C. The odor activity value (OAV), which indicates a compound’s contribution to the overall aroma, was calculated as the ratio of its concentration (C) to its OT. Detailed description was provided in the Supplementary Material.

### 2.11. Recombination and Omission Experiments

For aroma recombination, according to previous research, aroma compounds with OAVs ≥ 1 in each sample were added to odorless refined camellia oil based on their quantitative results to prepare the aroma recombination sample [25]. The respective recombinates were evaluated by the sensory panel as described above for APA. For omission experiments, triangle tests were carried out with one omitted sample and two recombinant samples according to previous research [19]. The omission experiment was conducted by removing either one odor compound from the complete recombination model. Twelve trained panelists (six males and six females) who can accurately identify aroma differences between four reconstituted models and the corresponding omission model were counted. Detailed description was provided in the Supplementary Material.

### 2.12. Statistical Analysis

All samples were tested in triplicate, and the results are expressed as mean ± standard deviation. Significance analysis of the data (*p* < 0.05) was carried out using SPSS 22. Cluster heat map was drawn using TBtools-II (v2.362). Histograms, partial least-squares regression (PLSR), sparse partial least-squares discriminant analysis (sPLS-DA) were drawn using Origin 2021, UnscramblerX 10.4, and MetaboAnalyst 6.0, respectively.

## 3. Results

### 3.1. Evolution of the Sensory Profile from Z. bungeanum Volatile Fractions

**Figure 1:**
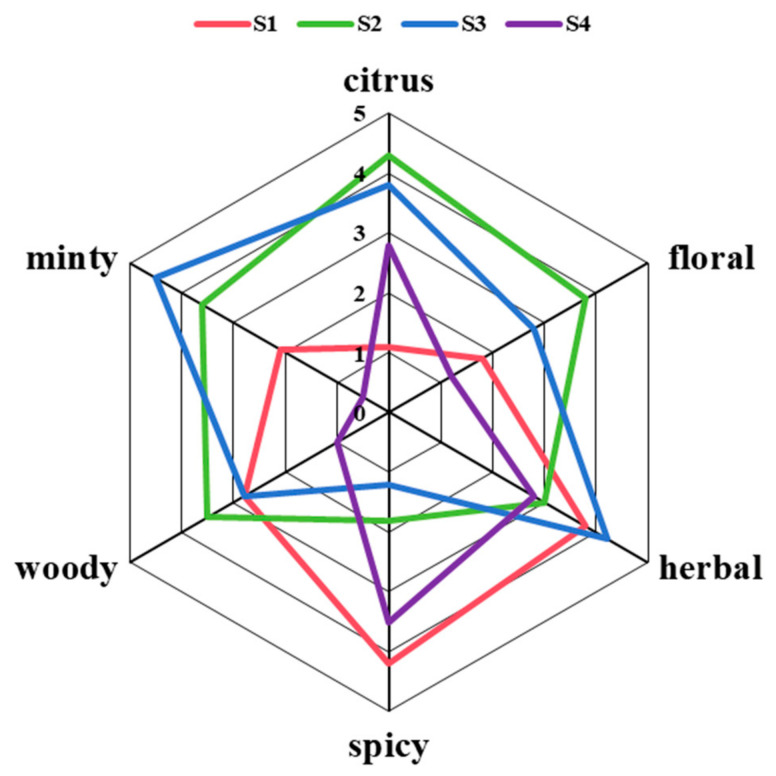
Sensory evaluation of *Z. bungeanum* volatile fractions from solvent-extracted pericarp VEs (S1), steam-distilled pericarp EOs (S2), steam-distilled leaf EOs (S3), and solvent-extracted leaf VEs (S4).

Odor attributes and descriptions of S1–S4 were evaluated using six representative odor attributes (citrus, floral, herbal, spicy, woody, and minty) as provided in Table S1. From Figure 1, distinct aromatic landscapes and intensity variations were observed across the different matrices and extraction methodologies. For the pericarp-derived fractions, the solvent-extracted pericarp VEs (S1) exhibited a dominant spicy and herbal character, achieving high intensity scores of 4.2 ± 0.6 and 3.8 ± 0.5, respectively, while presenting low scores for citrus (1.1 ± 0.2) and floral (1.8 ± 0.3) notes. In contrast, the steam-distilled pericarp EOs (S2) presented a highly robust and balanced profile, characterized by pronounced citrus (4.3 ± 0.5) and floral (3.8 ± 0.4) intensities, accompanied by notable minty (3.6 ± 0.4) and woody (3.5 ± 0.3) nuances, but a significantly lower spicy score (1.8 ± 0.2).

Regarding the leaf by-products, two divergent sensory directions emerged. The steam-distilled leaf EOs (S3) were defined by intense minty and herbal aromas, recording the highest scores among all fractions for these attributes at 4.5 ± 0.5 and 4.2 ± 0.4, respectively, alongside a moderate citrus note (3.8 ± 0.3). Conversely, the solvent-extracted leaf Ves (S4) displayed a flatter sensory profile with the lowest overall odor intensities across most attributes. It presented perceptible spicy (3.5 ± 0.3) and herbal (2.8 ± 0.3) nuances, but extremely weak floral (1.2 ± 0.1), woody (1.0 ± 0.2), and minty (0.5 ± 0.1) characteristics. These differences are fundamentally driven by the underlying extraction mechanisms [26].

Hierarchical clustering of E-nose data (Figure S1) corroborated these findings, clearly isolating S4 due to its low volatile intensity and positioning S2 and S3 closely, reflecting their shared distillation-derived markers. These differences, though subtle, were consistently captured by both the sensory evaluation and the instrumental response patterns, confirming the reliability of the integrated sensory–electronic approach. These sensory profiles established the foundational framework for the subsequent molecular identification of key odorants.

### 3.2. Identification of Aroma-Active Compounds in Z. bungeanum Volatile Fractions

**Figure 2:**
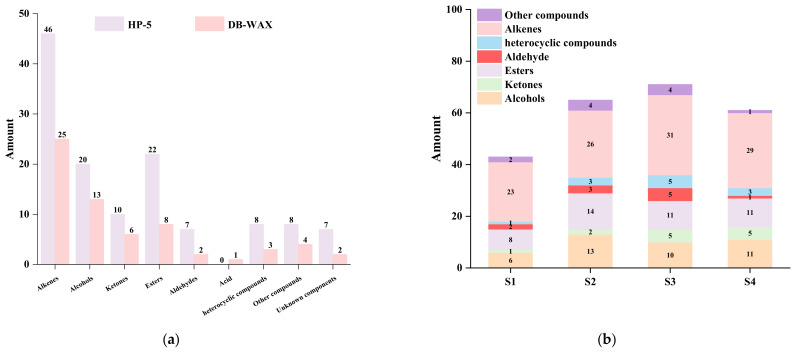
Qualitative analysis of *Z. bungeanum* volatile fractions (S1–S4): (**a**) Amount of odor compounds identified via GC-MS-O using DB-WAX and HP-5 columns. (**b**) Amount of different chemical classes of odorants by HP-5.

GC-MS-O was applied to determine the aroma landscapes of four distinct volatile fractions (S1–S4). Both non-polar (HP-5) and polar (DB-WAX) columns were utilized for comprehensive qualitative profiling (Table S3). On the HP-5 column, a total of 128 odor compounds were identified, including 46 alkenes, 20 alcohols, 10 ketones, 22 esters, 7 aldehydes, 8 heterocyclic compounds, 8 other compounds, and 7 unidentified components (Figure 2a). Conversely, the DB-WAX column [15] facilitated the identification of 64 volatiles, comprising 25 alkenes, 13 alcohols, 6 ketones, 8 esters, 2 aldehydes, 1 acid, 3 heterocyclic compounds, 4 other compounds, and 2 unknown components. These results reinforce that HP-5 columns provide a superior detection range for the terpene-rich molecular landscapes characteristic of the *Zanthoxylum* species [20]. Consequently, the HP-5 column was selected as the optimal stationary phase for the subsequent quantitative determination of aroma-active compounds owing to its significantly broader identification coverage and superior resolution. In addition, 62 aroma compounds were actively perceived through olfactometry, with their respective flavor dilution (FD) factors detailed in Table S3. Compared with previous profiling, this flavoromics approach demonstrated heightened sensitivity in distinguishing minor constituents, effectively bridging the chemical-sensory gap [16,27].

The qualitative profiling revealed that the number of volatile components varied significantly across the matrices and extraction methods (Figure 2b). Leaf-derived fractions demonstrated superior molecular diversity: steam-distilled leaves (S3) and solvent-extracted leaves (S4) yielded 71 and 68 volatiles, respectively, compared with 65 compounds detected in steam-distilled pericarps (S2) and 43 in solvent-extracted pericarps (S1). Specifically, leaf fractions (S3 and S4) were characterized by a higher abundance of alkenes (31 and 29, respectively) and alcohols (10 and 11, respectively). Furthermore, S4 featured seven unknowns that were absent in the pericarp-derived S1.

Regarding specific chemical classes, alkenes (nos. ***68***–***113***), mainly monoterpenes and sesquiterpenes, dominated the volatile landscape with 46 compounds identified, imparting woody, citrus, floral, and herbal notes. The number of alkenes in S3 (31) and S4 (29) surpassed those in S2 (26) and S1 (17). Notably, limonene (nos. ***75***) recorded an FD factor of 729 in S2 and 81 in S1, providing the foundational citrus aroma. Meanwhile, other alkenes (nos. ***70***, ***71***, ***73***, ***74***, ***86***) in S2, including sabinene, β-myrcene, α-phellandrene, α-terpinene, and caryophyllene, all recorded FD factors ≥ 27, illustrating the complex woody–citrus synergy unique to *Zanthoxylum*. Following alkenes, 22 esters (nos. ***38***–***59***) were identified, with both S3 and S4 exhibiting 11 distinct compounds each, surpassing the 8 types found in S1. Linalyl acetate (nos. ***40***) consistently showed high FD factors across all samples (FD = 81 in S1, S2, and S3; FD = 27 in S4), and 4-terpinenyl acetate (no. ***41***) reached its maximum potency in S2 (FD = 243), indicating its major role in defining floral, citrus, and woody richness [28]. Twenty alcohols (nos. ***18***–***37***) were also detected, among which S4 yielded 11 distinct compounds. Linalool (nos. ***19***) emerged with high FD factors (FD = 81 in S1, 729 in S2, and 27 in S4) as the fundamental molecular backbone for the fresh citrus–floral scent of *Zanthoxylum* [29]. In S4, 4-carvomenthenol, α-acorenol, and t-cadinol (nos. ***19***, ***32***, and ***33***) all presented an FD factor of 27, while α-terpineol (nos. ***22***) showed an FD factor of 27 in S2 and 9 in S3. Additionally, ten ketones (nos. ***8***–***17***) and seven aldehydes (nos. ***1***–***7***) were identified. Leaf-derived fractions (S3 and S4) each harbored 5 distinct ketones, exclusively including (E)-β-ionone (nos. ***15***), whereas carvone (nos. ***9***) showed an FD factor of 27 in S2 and 9 in S1. S3 also exhibited the highest variety of aldehydes (5 types), including decanal and undecanal (nos. ***2*** and ***5***). Cuminaldehyde (nos. ***3***), characterized by spicy and herbal notes, exhibited an FD factor of 27 in S1 and 1 in S4, while phellandral (nos. ***4***) was dominant in S2 (FD = 27), characterized by citrus–herbal tones. Furthermore, eight heterocyclic compounds (nos. ***60***–***67***) were detected; caryophyllene oxide (nos. ***62***) was present in all fractions (FD = 3 vs. 9 vs. 1 vs. 1), providing a persistent woody base note, and eucalyptol (nos. ***67***) reached its highest potency in S3 (FD = 27). Finally, eight additional trace compounds (nos. ***114***–***121***) showed a consistent presence across all four fractions, with (-)-β-elemene (nos. ***117***) recording an FD factor of 9 in S2, known for its antioxidant potential, suggesting possible biofunctional roles beyond aroma [6]. Other minor components displayed low FD values, contributing primarily to the overall aroma synergy. The identification results confirm the complex terpene-rich landscapes of the *Z. bungeanum* fractions, with leaf matrices demonstrating distinctly higher qualitative richness than traditional pericarps. Collectively, these qualitative profiles and FD factors establish the comprehensive aroma-active fingerprints of the four fractions, providing the essential chemical basis for the subsequent quantitative comparisons and sensory validations.

### 3.3. Quantitative Analysis and Odor Activity Value Calculation of Key Aroma Compounds in Z. bungeanum Volatile Fractions

**Figure 3:**
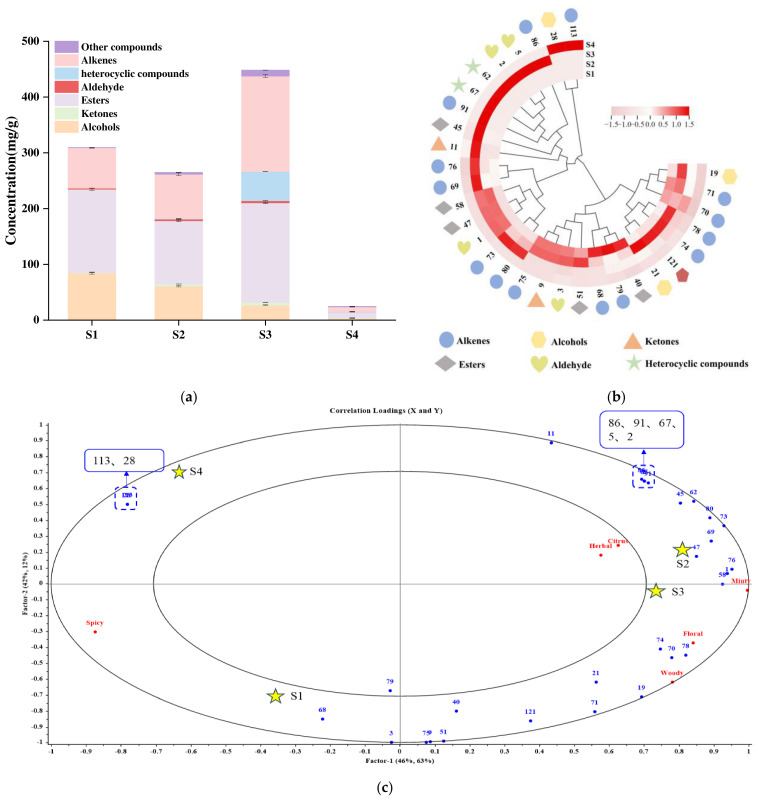
Quantitative analysis of *Z. bungeanum* volatile fractions (S1–S4): (**a**) Concentrations of different chemical classes of odorants by HP-5. (**b**) Cluster heatmap of key odorants (OAVs ≥ 1) across the four treatments. (**c**) Partial least-squares regression (PLSR) correlation loading plot illustrating the relationships between key odorants (X variables, OAVs) and sensory attributes (Y variables) across the four extraction treatments.

The strategic selection of extraction technologies and plant tissues induced pronounced variations in the quantitative concentration of aroma-active compounds (Table S4). While leaf-derived fractions exhibited superior qualitative richness compared with their pericarp counterparts, a striking discrepancy emerged when evaluating their quantitative landscapes (Figure 3a). The overall concentrations were predominantly governed by esters (149.76 vs. 113.43 vs. 178.48 vs. 10.51 mg/g for S1–S4, respectively), alkenes (72.50 vs. 79.38 vs. 171.09 vs. 8.37 mg/g), and alcohols (84.45 vs. 60.95 vs. 26.85 vs. 3.11 mg/g). These major classes were followed by heterocyclic compounds (0.002 vs. 1.07 vs. 52.54 vs. 0.53 mg/g), aldehydes (1.87 vs. 3.02 vs. 3.75 vs. 0.06 mg/g), ketones (0.52 vs. 3.81 vs. 4.95 vs. 1.19 mg/g), and others (0.41 vs. 0.32 vs. 10.78 vs. 0.88 mg/g). A sparse partial least-squares discriminant analysis (sPLS-DA) was executed to decode these chemical fingerprints (Figure S2). The resulting scores plot revealed a distinct spatial topology: the first principal component (PC1) decisively segregated the pericarp-derived fractions (S1, S2) from the leaf-derived matrices (S3, S4), whereas PC2 discriminated the samples based on the applied extraction methodology. The cluster heatmap of key odorants (OAVs ≥ 1) (Figure 3b) further revealed that pericarp fractions (S1, S2) were primarily composed of esters and alcohols, with their alkene fractions dominated by monoterpenes (54.02 vs. 60.03 mg/g). In stark contrast, S3 was defined by its exceptionally high content of esters (178.48 mg/g) and alkenes (171.09 mg/g), with the latter fraction being almost entirely composed of sesquiterpenes (137.06 mg/g). S3 also contained a uniquely high level of heterocyclic compounds (52.54 mg/g). At the individual compound level, S1 was distinguished by its high concentration of linalyl acetate (nos. ***40***, 120.82 mg/g), while S2 was rich in linalool (nos. ***19***, 17.56 mg/g) and limonene (nos. ***75***, 31.56 mg/g). The profile of S3 was driven by α-terpinyl acetate (nos. ***45***, 124.16 mg/g), eucalyptol (nos. ***67***, 49.75 mg/g), and caryophyllene (nos. ***86***, 45.56 mg/g). Conversely, S4 consistently yielded the lowest absolute concentrations across all chemical classes.

To translate these quantitative chemical variations into human sensory perception, odor activity values (OAVs) were calculated (Table 1). As illustrated in Figure 3b, a total of 31 key odorants with an OAV ≥ 1 were specifically screened from the 62 aroma-active compounds initially identified with a flavor dilution (FD) factor range of 1 to 729. The pericarp extract (S1) was characterized by a complex spicy–citrus–floral profile, primarily driven by (E)-β-ocimene (nos. ***76***, OAV = 83,853), linalool (nos. ***19***, OAV = 8353), linalyl acetate (nos. ***40***, OAV = 8055), β-myrcene (nos. ***71***, OAV = 4023), limonene (nos. ***75***, OAV = 2991), and cuminaldehyde (nos. ***3***, OAV = 778). Distilled pericarp EO (S2) exhibited a potent citrus–floral character, with an even stronger floral impact from (E)-β-ocimene (OAV = 107,588) and linalool (OAV = 13,509). S2 also displayed secondary notes of minty, primarily from carvone (nos. ***9***, OAV = 1106), and a strong woody character contributed by α-pinene (no. **69**, OAV = 5503), alongside detectable spicy odorants like β-myrcene (OAV = 4353) and cuminaldehyde (OAV = 873). The distilled leaf EO (S3) presented a powerful herbal–woody profile overwhelmingly dominated by (E)-β-ocimene (OAV = 189,971) and α-terpinyl acetate (nos. ***45***, OAV = 155,196), supported by eucalyptol (nos. ***67***, OAV = 49,753), α-pinene (OAV = 14,298), humulene (nos. ***91***, OAV = 6828), and caryophyllene (nos. ***86***, OAV = 4556). Finally, the solvent-extracted leaf VE (S4) showed the lowest overall odor activity, presenting a weaker herbal profile driven by α-terpinyl acetate (OAV = 5443), (E)-β-ocimene (OAV = 3324), and α-elemol (nos. ***28***, OAV = 2920), but exhibited a perceptible spicy impact driven by cuminaldehyde (OAV = 153) that was stronger than distilled EOs (S3).

To further reveal the multivariate relationships between odor attributes and key odorants, partial least-squares regression (PLSR) was applied (Figure 3c). The spicy attribute was strongly associated with cuminaldehyde (nos. ***3***), β-myrcene (nos. ***71***), and γ-terpinene (nos. ***78***). In contrast, linalool (nos. ***19***), α-pinene (nos. ***69***), and limonene (nos. ***75***) were positively correlated with citrus and minty notes. Woody and floral attributes were linked to geranyl acetate (nos. ***47***), caryophyllene (nos. ***86***), and caryophyllene oxide (nos. ***62***). The herbal note was strongly associated with humulene (nos. ***91***) and 4-carvomenthenol (nos. ***21***). From the PLSR loading plot, S1 and S3 clustered toward the spicy–woody quadrants, reflecting their high contents of monoterpene hydrocarbons and sesquiterpenes, while S2 and S4 were positioned near the citrus–minty quadrants, driven by oxygenated monoterpenes such as linalool and linalyl acetate. Collectively, the quantitative, OAV, and PLSR analyses demonstrate that while the leaf and pericarp fractions share a foundational qualitative aroma blueprint, their distinct sensory identities are fundamentally governed by the quantitative variations in specific terpenoids induced by the different extraction methodologies.

### 3.4. Complementary Volatile Profiling via HS-GC-IMS

In total, 73 signal peaks (including monomers, dimers, and trimers) were detected across S1–S4 by HS-GC-IMS, leading to the identification of 65 aroma compounds (Table S5). These comprised 10 aldehydes, 7 ketones, 9 alcohols, 3 acids, 14 esters, 7 heterocycles, 10 alkenes, 4 others, and 1 unknown compound. Unlike the highly variable qualitative profiles observed in GC-MS-O, the qualitative detection of these 65 compounds was uniform across all four fractions. This uniform qualitative distribution indicates that the distinct chemical fingerprints distinguishing the leaf by-products from the pericarps in the IMS analysis are driven entirely by significant quantitative variations, rather than the mere presence or absence of specific markers. Most aroma compounds, except for specific alcohols (nos. ***A20***–***21***), an ester (nos. ***A39***), and monoterpenes (nos. ***A56***, ***A60***–***61***, ***A64***, and ***A67***–***68***), were exclusively identified via HS-GC-IMS and were absent in the GC-MS-O analysis. The abundance of these volatiles varied markedly with both extraction technique and plant part. The pericarp extract (S1) and EO (S2) exhibited intense signal responses for monoterpenes and esters, while the leaf EO (S3) showed high levels of monoterpene terpenoids and heterocyclic compounds. In contrast, the leaf extract (S4) displayed an overall weaker signal intensity. This constrained volatile content in S4 aligns with its role as a milder baseline matrix for flavor mimicry, functioning without the overpowering sensory dominance of heavy terpenoids.

**Figure 4:**
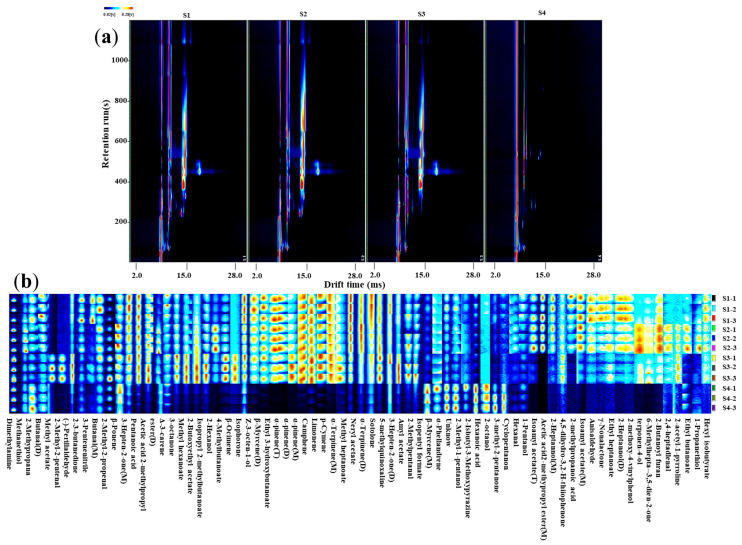
HS-GC-IMS volatile fingerprinting of *Z. bungeanum* fractions (S1–S4): (**a**) Top-view plot of signal intensities; (**b**) gallery plot of signal intensities.

The top-view plot (Figure 4a) illustrates that the aroma compounds in S1–S4 appeared at retention times between 50 and 1500 s, with more pronounced red signals indicating higher concentrations. The odor profiles of the four matrices were clearly distinguishable in the gallery plot (Figure 4b). Notably, several low-molecular-weight (C < 6) volatiles, such as 2-methyl-2-propenal (nos. ***A2***), isophorone (nos. ***A24***), and methyl acetate (nos. ***A33***), which impart floral or woody odors, were exclusively detected by GC-IMS. This highlights the superior sensitivity of GC-IMS for trace volatiles with high volatility or low polarity [30]. The compositional patterns further validated that leaf by-products possess qualities both overlapping with and distinct from those of the pericarp. S3 contained unique responses for (-)-perillaldehyde (nos. ***A9***) and α-terpinene (nos. ***A67***), strongly reinforcing its sensory innovation profile driven by minty–herbal notes. Conversely, cyclopentanone (nos. ***A28***, minty) was relatively abundant in S2, and (E)-3-pentenenitrile in S1 (nos. ***A71***, spicy). Furthermore, S4 showed limited detectable α-pinene (nos. ***A61***, herbal–woody), suggesting its loss during solvent extraction. These findings demonstrate the complementary nature of GC-IMS and GC-MS in profiling aroma complexity, proving that specific extraction methods can capture both the shared similarities and the unique differences between tissues.

Interestingly, GC-IMS also enabled the detection of trace off-flavor compounds such as 2-methylpropanoic acid (nos. ***A32***, rancid) and dimethylamine (nos. ***A69***, fishy), which escaped GC-MS detection due to their high volatility and small molecular mass. Similar observations have been reported in other matrices, such as microalgae and fermented products, where IMS shows enhanced sensitivity toward small polar compounds [30]. Most importantly, the presence of these trace off-notes in the leaf by-products did not detrimentally impact their overall sensory profile. Overall, the exclusive detection of low-molecular-weight volatiles (C < 6) and trace off-flavors by HS-GC-IMS, which were absent in the GC-MS-O analysis, demonstrates the complementary nature of these dual analytical platforms. The integrated use of specific extraction methods and dual detection systems successfully captured both the shared qualitative similarities and the unique quantitative differences across the different *Zanthoxylum* tissues.

### 3.5. Aroma Reconstitution and Omission Experiments

**Figure 5:**
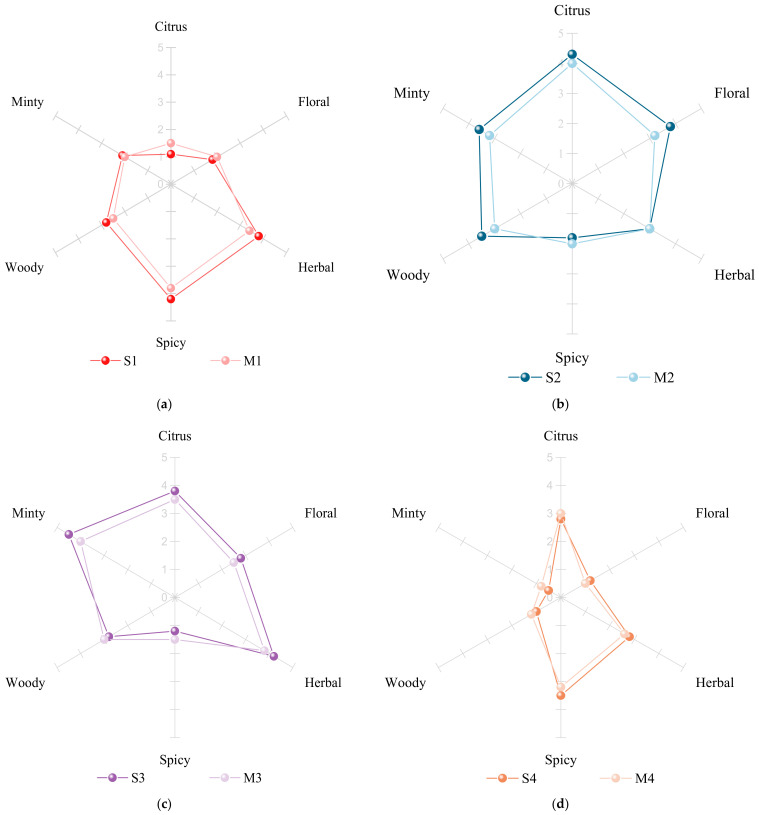
Radar charts comparing the sensory profiles of the original *Z. bungeanum* volatile fractions with their corresponding aroma recombination models: (**a**) S1 vs. M1; (**b**) S2 vs. M2; (**c**) S3 vs. M3; (**d**) S4 vs. M4.

To verify the contribution of individual key aroma compounds to overall odor perception, aroma recombination and omission experiments were performed using the key odorants with OAV ≥ 1. In total, 21 compounds (only dibutyl phthalate with an OAV < 1) were selected to reconstruct the aroma matrix of the four samples. As shown in Figure 5a–d, the radar charts of recombination models (M1–M4) exhibited a high degree of similarity to the original aroma of S1–S4, confirming that the identified key odorants reliably represent the characteristic aroma profiles. Omission tests further elucidated the individual contribution of each key odorant (Table 2). Significant sensory differences (*p* < 0.05) were observed for 20 compounds when excluded from the recombination model. The absence of cuminaldehyde (nos. ***3***), β-myrcene (nos. ***71***) and limonene (nos. ***75***) caused the most pronounced decrease in spicy and citrus notes in S1 and S2 (*p* < 0.001), confirming their dominant contribution to the pericarp-derived aromas. In contrast, S3 demonstrated a clear sensory departure from the pericarp, highlighting the leaf’s unique aromatic potential. The omission of caryophyllene (nos. ***86***) and humulene (nos. ***91***) led to a significant reduction (*p* < 0.01) in woody and herbal intensities in S3. Both compounds are sesquiterpene hydrocarbons synthesized via the MVA pathway through cyclization of farnesyl pyrophosphate by caryophyllene synthase [31]. These molecules often impart earthy and woody nuances in spices, consistent with their high OAVs and frequent detection in *Zanthoxylum* or *Piper* species [32]. Similarly, the removal of carvone (nos. ***9***), a monoterpene ketone derived from oxidation of limonene via (-)-carveol dehydrogenase, resulted in reduced minty notes in S2 and S3 (*p* < 0.01) [33]. Conversely, the relatively weak overall aroma intensity of S4 meant that removing neryl acetate (nos. ***58***) or limonene (nos. ***75***) only slightly decreased citrus freshness. This reflects the milder nature of S4, which serves as a clean slate for flavor mimicry. Although it lacks the overwhelming potency of pericarps, its retention of core markers like limonene and cuminaldehyde provides a subtle spicy–citrus framework that effectively mirrors the foundational character of commercial products without the interference of high-intensity floral notes.

Although linalool (nos. ***19***) and linalyl acetate (nos. ***40***) exhibited high OAVs and FD factors (FD ≥ 27, OAV ≥ 1), their omission resulted in moderate sensory changes (*p* < 0.05), suggesting a synergistic rather than dominant contribution, as described in a previous study [34]. Importantly, dibutyl phthalate (nos. ***52***, FD = 0, OAV < 1 in S1, undetected in S2–S4) had no significant sensory impact when omitted, confirming its non-contributory role and validating the rationale for selecting compounds with OAV ≥ 1 as true aroma-active substances, since only those above the odor threshold can perceptibly influence the overall aroma. Collectively, the omission data demonstrate that β-myrcene, limonene, caryophyllene, humulene, and carvone are the primary odorants shaping the characteristic aroma complexity of *Z. bungeanum* volatile fractions. The combination of sensory evaluation, recombination/omission validation, and PLSR modeling establishes that monoterpenes (limonene, β-myrcene) and sesquiterpenes (caryophyllene, humulene) are the core markers of this complex aroma landscape, consistent with biosynthetic patterns observed in other aromatic plants like *Citrus*, *Mentha*, and *Camellia* [35].

## 4. Discussion

### 4.1. Extraction Mechanisms Governing Volatile Release and Quantitative Profiles

The striking quantitative discrepancies observed among the *Z. bungeanum* fractions, particularly the low absolute volatile content in the leaf extract (S4) despite its rich qualitative diversity, stem directly from the underlying extraction mechanisms. Steam distillation (S2, S3) relies on steam volatility, effectively disrupting secretory cavities and rapidly liberating thermally stable monoterpenes and oxygenated terpenoids. The absence of solvent residues and the rapid vaporization process help preserve aroma integrity, yielding “true” EOs with high aromatic purity [36]. Conversely, solvent extraction (S1, S4) operates on the principle of “like dissolves like,” capturing a wider spectrum of heavier, lipophilic constituents. However, this often comes at the expense of the most volatile compounds due to evaporative losses during solvent removal [6]. Furthermore, the highly restricted compound release observed in S4 is fundamentally attributed to the dense cuticular structure of the leaves, which hinders solvent penetration and limits overall oil yield compared with the highly permeable pericarp tissues [37].

### 4.2. Mechanistic Insights into Flavor Formation, Mimicry, and Sensory Innovation

The distinct sensory landscapes of *Z. bungeanum* fractions are fundamentally driven by specific biosynthetic pathways and processing-induced chemical transformations. Core terpenoid hydrocarbons (e.g., limonene, β-myrcene, caryophyllene, humulene), synthesized via the MEP/MVA pathways [31], dictate the foundational woody–citrus synergy. Processing significantly reconstructs this profile: for instance, the minty contributor carvone is derived from limonene oxidation [33], while the exclusive presence of (E)-β-ionone in leaf fractions originates from the targeted oxidative cleavage of leaf pigments like β-carotene [38].

Translating these chemical concentrations into odor activity values (OAVs) reveals critical perceptual interactions, namely sensory masking and unmasking effects, that dictate the upcycling potential of leaf by-products. In the pericarp EO (S2), although spicy odorants were detectable, their perception was suppressed by the extremely high OAVs of floral and citrus compounds, creating a potent sensory masking effect. Conversely, a sensory unmasking effect was distinctly observed in the solvent-extracted leaf (S4). The absence of overpowering floral or citrus interferents allowed core markers like limonene and cuminaldehyde to bridge the sensory gap, enabling the milder S4 matrix to effectively mimic the foundational spicy–citrus character of traditional pericarp products. Beyond flavor mimicry, steam distillation of leaves (S3) unlocks a powerful sensory innovation. Driven by MVA-pathway derivatives (caryophyllene and humulene) and eucalyptol [32], S3 introduces robust herbal–woody and minty dimensions that are fundamentally absent in premium pericarp EOs.

Furthermore, while highly sensitive GC-IMS analysis revealed trace native off-flavors in the leaf by-products (e.g., dimethylamine), the overwhelming abundance and high OAVs of the primary terpenoids and esters likely dominate the overall human olfactory perception, perceptually overshadowing these minor background notes. This perceptual dominance supports the seamless upcycling potential of leaf waste, allowing it to transcend its baseline to yield valuable and highly intense aromatic dimensions.

### 4.3. Contributions to Food Sustainability, Study Limitations, and Future Perspectives

The valorization of *Z. bungeanum* leaf by-products provides a robust framework for advancing sustainable food practices and circular economy initiatives. By transforming frequently discarded agricultural waste into versatile flavor resources, the industry can achieve both functional equivalence and novel sensory experiences, significantly reducing the environmental footprint of spice production.

However, two inherent limitations of the current study must be acknowledged. First, from a methodological perspective, although the aroma recombination models exhibited a high degree of similarity to the original profiles, there were constraints regarding commercial standard availability. Specifically, while 62 aroma-active compounds were initially identified within the FD range of 1–729, and 31 of these were screened as key odorants with an OAV ≥ 1, only 20 true key aroma compounds were practically available for inclusion in the reconstitution and omission experiments. This constraint implies that some minor synergistic interactions shaping the complex matrix might not be fully captured. Second, from an industrial perspective, this chemical variance was highly consistent with the macro extraction yields, which were determined to be 2.1%, 1.7%, 0.2%, and 1.9% for S1–S4, respectively. The absolute volatile yield and extraction efficiency of leaf matrices, particularly during solvent extraction, remain notably lower than those of pericarp counterparts due to the aforementioned cuticular resistance.

Despite these limitations, the present study firmly establishes the profound industrial value of leaf valorization. The comprehensive identification of robust quality markers, specifically key monoterpenes (β-myrcene, limonene) and sesquiterpenes (caryophyllene, humulene), provides reliable molecular targets for quality control. Future research should prioritize optimizing green extraction techniques (e.g., enzyme-assisted or supercritical fluid extraction) to overcome the yield limitations of leaf cuticles and evaluate the stability of these upcycled flavor ingredients within complex food matrices during thermal processing and prolonged storage.

## 5. Conclusions

In conclusion, this study successfully decoded the aromatic potential of *Z. bungeanum* leaf by-products using an integrated sensomics approach. Moving beyond traditional pericarp utilization, our findings confirm that the frequently discarded leaf matrix harbors a remarkably complex aromatic reservoir that exhibits superior molecular diversity compared with its pericarp counterparts. Crucially, this study demonstrates that targeted extraction technologies can dynamically tailor this volatile reservoir to fulfill dual industrial roles. On one hand, solvent extraction allows leaf by-products to achieve aromatic parity with pericarps, effectively replicating the foundational spicy–citrus profile for flavor mimicry. On the other hand, steam distillation unlocks a distinctive herbal–woody sensory innovation, hallmarked by eucalyptol and complex ketones, that expands the aromatic boundaries of traditional spices. Furthermore, sensomics validation conclusively identified β-myrcene, limonene, caryophyllene, and humulene as the core quality markers dictating these perceptual shifts. Ultimately, this research provides a robust theoretical foundation for the upcycling of *Z. bungeanum* agricultural waste. By delivering both functional equivalence and novel sensory experiences, these findings facilitate the transition toward a circular bio-economy and offer the global food industry a sustainable, customizable source of premium flavoring ingredients.

## Figures and Tables

**Table 1:** Odor thresholds (OTs) and odor activity values (OAVs) of odor compounds identified in S1–S4.

NO.	Name	OTs (μg/kg)	OAV			
			S1	S2	S3	S4
**Aldehyde**						
1	(+)-Citronellal ^b^	25	n.d.	17,840	18,560	n.d.
2	Decanal ^b^	650	n.d.	n.d.	1629	n.d.
3	Cuminaldehyde ^a^	400	778	873	n.d.	153
5	Undecanal ^b^	5000	n.d.	n.d.	134	n.d.
**Ketones**						
9	Carvone ^a^	500	1044	1106	n.d.	n.d.
11	Piperitone ^a^	1200	n.d.	n.d.	369	138
**Alcohols**						
19	Linalool ^a^	1300	8353	13,509	7425	578
21	4-Carvomenthenol ^a^	2000	1009	5178	1301	<1
28	α-Elemol ^b^	100	n.d.	n.d.	n.d.	2920
**Esters**						
40	Linalyl acetate ^a^	15,000	8055	4574	2765	290
45	α-Terpinyl acetate ^a^	800	27,346	32,960	155,196	5443
47	Geranyl acetate ^a^	10,000	n.d.	216	216	<1
51	Methyl palmitate ^a^	1000	81	119	n.d.	n.d.
58	Neryl acetate ^a^	2000	113	280	270	111
**Heterocyclic compounds**						
62	Caryophyllene oxide ^a^	4000	<1	<1	90	<1
67	Eucalyptol ^b^	1000	n.d.	n.d.	49,753	n.d.
**Alkene**						
68	α-Thujene ^b^	4400	279	140	<1	<1
69	α-Pinene ^a^	400	5045	5503	14,298	n.d.
70	Sabinene ^b^	1000	7715	7282	7815	258
71	β-Myrcene ^a^	600	4023	4353	2522	n.d.
73	α-Phellandrene ^b^	160	n.d.	2100	4225	n.d.
74	α-Terpinene ^b^	10,000	<1	353	172	<1
75	Limonene ^a^	10,000	2991	3156	n.d.	<1
76	trans-β-Ocimene ^b^	34	83,853	107,588	189,971	3324
78	γ-Terpinene ^a^	2000	478	1331	827	114
79	Terpinolene ^a^	4000	1200	375	289	<1
80	Alloocimene ^b^	4000	n.d.	112	192	<1
86	Caryophyllene ^a^	10,000	<1	<1	4556	121
91	Humulene ^a^	2000	445	442	6828	327
113	Styrene ^a^	800	n.d.	n.d.	n.d.	148
**Others**						
121	o-Cymenea ^a^	250	288	612	84	n.d.

Odor thresholds (OTs) in oil: ^a^ Newly determined in this study; ^b^ The threshold of volatile compounds in water referred to in the literature. Compilations of Odor Threshold Values in Air, Water and Other Media [9]. n.d. means that the compound was not detected.

**Table 2:** Omission models for odor compounds in *Z. bungeanum* volatile fractions (S1–S4).

NO.	Omitted Compound	Significance			
		S1	S2	S3	S4
3	Cuminaldehyde	***	*	NS	*
9	Carvone	**	**	NS	NS
11	Piperitone	NS	NS	*	*
19	Linalool	*	***	NS	NS
21	4-Carvomenthenol	**	*	*	NS
40	Linalyl acetate	*	**	**	*
45	α-Terpinyl acetate	**	**	***	**
47	Geranyl acetate	NS	*	**	*
51	Methyl palmitate	NS	*	NS	NS
52	Dibutyl phthalate	NS	NS	NS	NS
58	Neryl acetate	*	*	*	*
62	Caryophyllene oxide	NS	NS	**	NS
69	α-Pinene	**	*	**	
71	β-Myrcene	***	**	***	NS
75	Limonene	**	**	NS	***
78	γ-Terpinene	*	**	*	**
79	Terpinolene	*	**	*	*
86	Caryophyllene	NS	*	***	NS
91	Humulene	NS	*	***	*
113	Styrene	NS	NS	NS	NS
121	o-Cymene	*	NS	NS	NS

“*” Indicated significance at *p* < 0.05; “**” indicated significant at *p* < 0.01; “***” indicated significant at *p* < 0.001; NS: no significant difference.

## Data Availability

The original contributions presented in this study are included in the article/Supplementary Material. Further inquiries can be directed to the corresponding authors.

## Supplementary Materials

The following supporting information can be downloaded at: https://www.mdpi.com/article/10.3390/foods15122243/s1. Table S1. The odor attributes, corresponding odor descriptors, and reference odor compounds used in sensory evaluation. Table S2. Linear calibration curves and response factors (Rf) of external standard used in the quantitative determination of the aroma compounds by GC-MS in SIM mode. Table S3. Retention indices (RI) and odor descriptions of odor compounds identified in *Z. bungeanum* volatile fractions via GC-MS-O with S1–S4. Table S4. GC-MS quantification of *Z. bungeanum* volatile fractions from solvent-extracted pericarp extracts (S1), steam-distilled pericarp essential oils (S2), steam-distilled leaf essential oils (S3), and solvent-extracted leaf extracts (S4). Table S5. HS-GC-IMS identification of *Z. bungeanum* volatile fractions from solvent-extracted pericarp extracts (S1), steam-distilled pericarp essential oils (S2), steam-distilled leaf essential oils (S3), and solvent-extracted leaf extracts (S4). Figure S1. Cluster heat map of *Z. bungeanum* volatile fractions from S1–S4 via E-nose. Figure S2. sPLS-DA analysis of *Z. bungeanum* volatile fractions from S1–S4. Reference [39] is cited in the supplementary materials.
