# Supplementary material for: Valorization of Zanthoxylum bungeanum Maxim. Leaf By-Products: Comparative Aroma Profiling with Pericarps Across Extraction Strategies
Source: Foods. 2026 Jun 22;15(12):2243. doi: 10.3390/foods15122243 (PMC13298970; doi:10.3390/foods15122243)
Supplement: Supplementary file 1 [file foods-15-02243-s001.zip › foods-4338323-supplementary.pdf]

Table S1. The odor attributes, corresponding odor descriptors, and reference odor compounds used in sensory evaluation.

Sensory attributes	Description	Reference compound*
Floral	Sweet, rosy, lavender-like, jasmine-like, powdery	Linalool
minty	minty, cooling, camphoraceous, mentholic, peppermint-like, fresh, herbal-green	Carvone
Citrus	Lemon, orange, lime, grapefruit, fresh, zesty	Limonene
Spicy	Peppery, clove-like, cinnamon, pungent, warm	Cuminaldehyde
Woody	Cedar, pine, earthy, balsamic, resinous	α-Pinene
Herbal	Minty, camphoraceous, grassy, leafy, fresh herbs	Linalyl acetate

*The reference compounds were identified by this study and checked with the literature[1-4].

Table S2. Linear calibration curves and response factors (Rf) of external standard used in the quantitative determination of the aroma compounds by GC-MS in SIM mode.

No.[a]	Identified odorants	Selected ions (m/z) [b]	Calibration curve equation [c]	linear range (mg/mL)	R^2 [d]	Recovery (%)	Rf [e]
3	Cuminaldehyde	133	y = 0.3856x - 0.0222	0.02-0.04	0.9922	90.1	0.864
9	Carvone	82	y = 1.2418x - 0.4389	0.05-0.1	0.99903	114.69	0.971
11	Piperitone	82	y = 0.1761x - 0.0025	0.005-0.01	0.99339	112.73	1.216
19	Linalool	71	y = 0.3935x + 0.0275	0.04-0.1	0.9982	87.72	0.954
21	4-Carvomenthenol	71	y = 0.4941x + 0.0127	0.04-0.1	0.99902	92.66	0.978
40	Linalyl acetate	93	y = 0.2178x + 0.1089	0.05-0.1	0.9923	83.42	1.112
45	α-Terpinyl acetate	121	y = 0.1645x + 0.0045	0.008-0.02	0.9958	113.68	0.865
47	Geranyl acetate	69	y = 0.9275x - 0.4596	0.01-0.02	0.9904	106.53	0.9895
51	Methyl palmitate	74	y = 0.3033x - 0.0079	0.01-0.02	0.9941	87.32	0.879
52	Dibutyl phthalate	149	y = 5.3307x - 0.003	0.01-0.02	0.9999	94.11	0.897
58	Neryl acetate	69	y = 3.2286x - 0.6289	0.02-0.05	0.995	115.66	0.763
62	Caryophyllene oxide	43	y = 4.0756x - 0.44	0.02-0.04	0.9921	83.45	0.878
69	α-Pinene	93	y = 0.0346x + 0.001	0.02-0.04	0.9988	104.55	0.98
71	β-Myrcene	41	y = 0.4464x + 0.005	0.02-0.04	0.9964	117.61	0.957
75	Limonene	68	y = 0.2904x + 0.0127	0.02-0.05	0.9967	92.71	1.122
78	γ-Terpinene	93	y = 0.4037x - 0.0901	0.01-0.02	0.9902	110.91	0.791
79	Terpinolene	93	y=0.2567x + 0.0032	0.01-0.02	0.9908	83.72	1.124
86	Caryophyllene	93	y = 0.5983x - 0.0137	0.008-0.02	0.9998	118.37	0.998
91	Humulene	93	y = 0.3301x + 0.0069	0.008-0.02	0.9905	81.69	0.964
113	Styrene	91	y = 1.7648x - 0.0059	0.002-0.004	0.9934	105.33	1.026
121	o-Cymene	119	y = 2.7536x + 0.0187	0.001-0.002	0.9946	94.67	0.971

[a]Aroma compounds can be sniffed via GC-MS-O. [b] Selected ions for target compound used for response factor and quantitation. [c] Calibration curves were constructed by the following formula: Ax/Ai=a(Cx/Ci) +b. A and C means the peak area and the concentration, while x and i means the external standard and internal standard, respectively. [d] R^2 values for linear regression of calibration curves. [e] Rf: response factors.

Table S3. Retention indices (RI) and odor descriptions of odor compounds identified in *Z. bungeanum* volatile fractions via GC-MS-O with S1-S4.

NO.	Name	RI[a]		Odor description	FD factors				Previously reported[b]			identification[c]
		HP-5	DB-WAX		S1	S2	S3	S4	A	B	C	
**Aldehyde**												
1	(+)-citronellal	1154	n.d.	herbal, citrus	n.d.	3	n.d.	n.d.	/	/	/	MS,RI,O
2	Decanal	1204	n.d.	waxy, citrus, fatty	n.d.	n.d.	3	n.d.	/	/	/	MS,RI,O
3	Cuminaldehyde	1237	n.d.	spicy, green, herbal	27	27	n.d.	1	/	/	/	MS,RI,O,S
4	Phellandral	1271	n.d.	citrus, herbal	n.d.	27	n.d.	n.d.	/	/	/	MS,RI,O
5	Undecanal	1305	n.d.	waxy, floral, aldehydic, citrus	n.d.	n.d.	1	n.d.	/	/	/	MS,RI,O
6	Z,Z-10,12-Hexadecadienal	1665	n.d.	waxy, oily	n.d.	n.d.	n.d.	n.d.	/	/	/	MS,RI
7	Pentadecanal	1710	n.d.	waxy	n.d.	n.d.	n.d.	n.d.	/	/	/	MS,RI
**Ketones**												
8	3,5-Octadien-2-one	1073	n.d.	fruity, fatty	n.d.	n.d.	n.d.	n.d.	/	/	/	MS,RI
9	Carvone	1242	n.d.	minty	9	27	n.d.	n.d.	/	/	√	MS,RI,O,S
10	2-Isopropyl-5-methyl-3-cyclohexen-1-one	1251	n.d.	minty, herbal, woody	n.d.	3	n.d.	n.d.	/	/	/	MS,RI,O
11	Piperitone	1252	n.d.	herbal, minty	n.d.	n.d.	n.d.	n.d.	/	/	/	MS,RI,S
12	Ionone	1273	n.d.	violet, sweet, floral, woody	n.d.	n.d.	1	n.d.	/	/	/	MS,RI,O
13	2-Undecanone	1292	n.d.	waxy, fruity, ketonic, fatty	n.d.	n.d.	n.d.	n.d.	√	√	/	MS,RI
14	1-(3,6,6-Trimethyl-1,6,7,7a-tetrahydrocyclopenta[c]pyran-1-yl)ethanone	1396	1781	fruity, sweet, woody	n.d.	n.d.	n.d.	n.d.	/	/	/	MS,RI
15	(E)-β-Ionone	1482	n.d.	floral, fruity, woody	n.d.	n.d.	n.d.	1	/	/	/	MS,RI,O
16	Hexahydrofarnesyl acetone	1852	n.d.	oily, herbal, woody	n.d.	n.d.	n.d.	n.d.	/	/	/	MS,RI
17	(E,E)-Farnesyl acetone	1916	n.d.	fruity, floral, woody, green	n.d.	n.d.	n.d.	n.d.	/	/	/	MS,RI
**Alcohols**												
18	cis-β-Terpineol	1068	n.d.	floral, citrus, woody, herbal,	n.d.	n.d.	n.d.	n.d.	/	/	/	MS,RI
19	Linalool	1100	1500	citrus, floral, woody	81	729	n.d.	27	/	/	/	MS,RI,O,S

Table S3 (continued)

NO.	Name	RI[1]		Odor description	FD factors				Previously reported			identification
		HP-5	DB-WAX		S1	S2	S3	S4	A	B	C	
20	trans-para-2-Menthen-1-ol	1120	1580	minty, herbal	n.d.	n.d.	n.d.	n.d.	/	/	/	MS,RI
21	4-Carvomenthenol	1175	1551	woody, mentholic, citrus, terpenic, spicy	27	27	3	n.d.	/	/	/	MS,RI,O,S
22	α-Terpineol	1190	n.d.	terpenic, citrus, woody, floral	n.d.	27	9	n.d.	/	/	/	MS,RI,O
23	γ-Terpineol	1216	n.d.	pine, floral, lilac	n.d.	n.d.	n.d.	n.d.	/	/	/	MS,RI
24	(Z)-Piperitol	1232	n.d.	herbal	n.d.	9	n.d.	n.d.	/	/	/	MS,RI,O
25	Plinol C	1232	n.d.	floral, woody, herbal	n.d.	n.d.	9	n.d.	/	/	/	MS,RI,O
26	5-Caranol, (1S,3R,5S,6R)-(-)-	1330	n.d.	woody, herbal, minty	n.d.	n.d.	n.d.	n.d.	/	/	/	MS,RI
27	4-epi-Cubebol	1499	n.d.	woody, minty, herbal	n.d.	n.d.	n.d.	n.d.	/	/	/	MS,RI
28	α-Elemol	1548	n.d.	green, woody, spicy, rose	n.d.	n.d.	n.d.	n.d.	/	/	/	MS,RI
29	Nerolidol	1563	n.d.	floral	n.d.	n.d.	n.d.	n.d.	/	/	/	MS,RI
30	7-epi-cis-sesquisabinene hydrate	1594	1361	woody, herbal	n.d.	1	n.d.	n.d.	/	/	/	MS,RI,O
31	Cubenol	1621	n.d.	spicy, herbal	n.d.	9	n.d.	n.d.	/	/	/	MS,RI,O
32	α-Acorenol	1629	n.d.	fruity, sweet, floral	n.d.	27	n.d.	n.d.	/	/	/	MS,RI,O
33	T-Cadinol	1638	n.d.	balsamic, earthy	n.d.	27	n.d.	n.d.	/	/	/	MS,RI,O
34	α-Cadinol	1651	n.d.	herbal, woody	n.d.	9	n.d.	n.d.	/	/	/	MS,RI,O
35	7-epi-trans-sesquisabinene hydrate	1682	n.d.	woody, herbal	n.d.	27	n.d.	n.d.	/	/	/	MS,RI,O
36	α-Bisabolol	1682	n.d.	floral, peppery, balsamic	n.d.	n.d.	n.d.	n.d.	/	/	/	MS,RI
37	5-Hydroxymethyl-1,1,4a-trimethyl-6-methylenedecahydronaphthalen-2-ol	1735	n.d.	woody	n.d.	n.d.	n.d.	n.d.	/	/	/	MS,RI
**Esters**												
38	Heptyl acetate	1114	n.d.	Green, waxy, citrus, woody	n.d.	n.d.	n.d.	n.d.	/	/	/	MS,RI
39	Octyl acetate	1212	n.d.	green, earthy, herbal	n.d.	n.d.	n.d.	n.d.	/	/	/	MS,RI
40	Linalyl acetate	1259	1514	herbal, citrus, sweet	81	81	81	27	√	√	√	MS,RI,O,S
41	4-Terpinenyl acetate	1296	n.d.	floral, citrus, woody	3	243	n.d.	n.d.	/	/	/	MS,RI,O

Table S3 (continued)

NO.	Name	RI[1]		Odor description	FD factors				Previously reported			identification
		HP-5	DB-WAX		S1	S2	S3	S4	A	B	C	
42	Isopulegyl acetate	1313	n.d.	woody, sweet, peppermint	n.d.	81	1	n.d.	/	/	/	MS,RI,O
43	Myrtenyl acetate	1322	n.d.	herbal, fruity, citrus	n.d.	n.d.	27	n.d.	/	/	/	MS,RI,O
44	2-Acetoxy-1,8-cineole	1340	n.d.	minty, clean, fresh, eucalyptus	n.d.	n.d.	n.d.	n.d.	/	/	/	MS,RI
45	α-Terpinyl acetate	1347	1651	herbal, citrus, spicy, woody, floral	9	81	3	1	/	/	/	MS,RI,O,S
46	Citronellyl acetate	1354	n.d.	floral, green, fruity, citrus, woody	1	n.d.	1	n.d.	/	/	/	MS,RI,O
47	Geranyl acetate	1383	1706	floral, rose, waxy, herbal	n.d.	27	9	1	/	/	/	MS,RI,O,S
48	Geranyl isovalerate	1603	n.d.	oily, herbal, fruity	n.d.	n.d.	n.d.	n.d.	/	/	/	MS,RI
49	Butyl octyl phthalate	1873	n.d.	faint	n.d.	n.d.	n.d.	n.d.	/	/	/	MS,RI
50	Methyl 11-hexadecenoate	1900	n.d.	waxy, fatty, oily	n.d.	n.d.	n.d.	n.d.	/	/	/	MS,RI
51	Methyl palmitate	1927	n.d.	oily, waxy, fatty	n.d.	n.d.	n.d.	n.d.	/	/	/	MS,RI,S
52	Dibutyl phthalate	1964	n.d.	faint	n.d.	n.d.	n.d.	n.d.	/	/	/	MS,RI,S
53	Methyl 8-octadecenoate	2096	n.d.	waxy, fatty, oily	n.d.	n.d.	n.d.	n.d.	/	/	/	MS,RI
54	Ethyl 9α-linolenate	2167	n.d.	oily, green, waxy	n.d.	n.d.	n.d.	n.d.	/	/	/	MS,RI
55	Dihydroactinidiolide	1523	n.d.	fruity, woody	n.d.	n.d.	n.d.	n.d.	/	/	/	MS,RI
56	Bornyl acetate	1282	n.d.	sweet, balsamic, woody, herbal	n.d.	27	n.d.	n.d.	/	/	/	MS,RI,O
57	Carvyl acetate	1362	n.d.	green, minty, herbal	n.d.	n.d.	27	n.d.	/	/	/	MS,RI,O
58	Neryl acetate	1365	1678	floral, citrus	3	27	n.d.	1	/	/	/	MS,RI,O,S
59	Farnesyl acetate	1849	n.d.	green, floral, waxy, citrus	n.d.	n.d.	n.d.	n.d.	/	/	/	MS,RI
**Heterocyclic compounds**												
60	2H-1-Benzopyran, 3,4,4a,5,6,8a-hexahydro-2,5,5,8a-tetramethyl-,	1287	n.d.	woody, earthy	n.d.	n.d.	1	n.d.	/	/	/	MS,RI,O
61	Manoyl oxide	2005	n.d.	woody, balsamic, earthy	n.d.	n.d.	n.d.	n.d.	/	/	/	MS,RI
62	Caryophyllene oxide	1574	1905	sweet, woody, spicy	3	9	1	1	/	/	/	MS,RI,O,S
63	Ledene oxide	1584	n.d.	woody, herbal, spicy	n.d.	n.d.	n.d.	n.d.	/	/	/	MS,RI

Table S3 (continued)

NO.	Name	RI[1] HP-5	RI[1] DB-WAX	Odor description	FD factors S1	S2	S3	S4	Previously reported A	B	C	identification
64	cis-Z-α-Bisabolene epoxide	1590	1800	woody, balsamic, sweet, herbal	n.d.	n.d.	n.d.	n.d.	/	/	/	MS,RI
65	Diepicedrene-1-oxide	1599	n.d.	woody, balsamic, herbal	n.d.	n.d.	n.d.	n.d.	/	/	/	MS,RI
66	Isoaromadendrene epoxide	1600	n.d.	woody, herbal, balsamic	n.d.	n.d.	n.d.	n.d.	/	/	/	MS,RI
67	Eucalyptol	1029	1184	herbal, minty	n.d.	n.d.	27	n.d.	/	/	/	MS,RI,O
**Alkene**												
68	α-Thujene	925	1005	woody, green, herbal	n.d.	n.d.	n.d.	n.d.	/	/	/	MS,RI
69	α-Pinene	931	n.d.	woody, terpenic, herbal	n.d.	9	27	n.d.	√	/	√	MS,RI,O,S
70	Sabinene	972	1096	woody, terpenic, citrus	1	27		n.d.	/	/	/	MS,RI,O
71	β-Myrcene	991	n.d.	terpenic, herbal, woody	9	27	1	n.d.	√	√	√	MS,RI,O,S
72	β-Pinene	993	1084	woody, piney, minty	n.d.	n.d.	n.d.	n.d.	/	/	√	MS,RI
73	α-Phellandrene	1000	n.d.	citrus, herbal, terpenic, green	n.d.	27	n.d.	n.d.	/	/	/	MS,RI,O
74	α-Terpinene	1014	1155	citrus, woody, terpenic	1	27	n.d.	n.d.	/	/	/	MS,RI,O
75	Limonene	1027	1175	citrus	81	729	n.d.	3	√	/	√	MS,RI,O,S
76	(E)-beta-Ocimene	1040	n.d.	sweet, herbal	n.d.	3	n.d.	n.d.	/	/	/	MS,RI,O
77	β-Ocimene	1050	n.d.	citrus, green, terpenic, woody	1	9	n.d.	n.d.	/	/	/	MS,RI,O
78	γ-Terpinene	1058	1220	oily, woody, terpenic, citrus	1	9	1	n.d.	/	/	/	MS,RI,O,S
79	Terpinolene	1085	1255	herbal, woody, citrus	n.d.	1	1	3	/	/	/	MS,RI,O,S
80	Alloocimene	1130	n.d.	floral, peppery, herbal	n.d.	3	n.d.	n.d.	/	/	/	MS,RI,O
81	para-Menthatriene	1138	n.d.	terpenic, herbal, woody	n.d.	n.d.	1	n.d.	/	/	/	MS,RI,O
82	1,5,5-Trimethyl-6-methylene-cyclohexene	1332	1340	citrus, herbal, woody	n.d.	n.d.	n.d.	n.d.	/	/	/	MS,RI
83	Copaene	1369	n.d.	woody, spicy	n.d.	9	n.d.	3	/	/	/	MS,RI,O
84	β-Bourbonene	1378	n.d.	herbal, woody, floral	n.d.	n.d.	n.d.	n.d.	/	/	/	MS,RI
85	α-Gurjunene	1400	n.d.	woody, balsamic	n.d.	n.d.	n.d.	n.d.	/	/	/	MS,RI

Table S3 (continued)

NO.	Name	RI[1]		Odor description	FD factors				Previously reported			identification
		HP-5	DB-WAX		S1	S2	S3	S4	A	B	C	
86	Caryophyllene	1410	1571	sweet, woody, spicy	3	81	1	1	/	/	/	MS,RI,O,S
87	β-Cubebene	1421	n.d.	citrus, fruity	n.d.	n.d.	n.d.	n.d.	/	/	/	MS,RI
88	β-Copaene	1421	n.d.	woody	n.d.	n.d.	n.d.	n.d.	/	/	/	MS,RI
89	γ-Elemene	1428	1604	green, woody, oily	n.d.	9	9	1	/	√	/	MS,RI,O
90	β-Ylangene	1437	n.d.	woody, sweet, balsamic, floral	n.d.	n.d.	n.d.	n.d.	/	/	/	MS,RI
91	Humulene	1445	1639	woody	27	27	1	n.d.	/	/	√	MS,RI,O,S
92	Alloaromadendrene	1452	n.d.	woody	n.d.	27	n.d.	n.d.	/	/	/	MS,RI,O
93	(+)-Calarene	1455	n.d.	woody, earthy	n.d.	n.d.	9	n.d.	/	/	/	MS,RI,O
94	Cedrene	1455	n.d.	woody	n.d.	9	n.d.	n.d.	/	/	/	MS,RI,O
95	α-Elemene	1467	n.d.	woody, spicy, herbal	n.d.	n.d.	n.d.	n.d.	/	/	/	MS,RI
96	γ-Muurolene	1470	1677	herbal, woody, spicy	n.d.	27	1	n.d.	/	/	/	MS,RI,O
97	Germacrene D	1473	n.d.	woody, spicy	1	27	n.d.	n.d.	/	/	/	MS,RI,O
98	Valencene	1478	n.d.	citrus, woody	n.d.	n.d.	1	n.d.	/	/	/	MS,RI,O
99	β-Selinene	1478	n.d.	herbal	n.d.	n.d.	n.d.	n.d.	/	/	/	MS,RI
100	Bicyclosesquiphellandrene	1483	n.d.	woody, earthy, herbal	n.d.	n.d.	n.d.	n.d.	/	/	/	MS,RI
101	α-Selinene	1487	n.d.	amber	n.d.	n.d.	n.d.	n.d.	/	/	/	MS,RI
102	Eremophilene	1488	n.d.	woody, earthy	n.d.	n.d.	1	n.d.	/	/	/	MS,RI,O
103	α-Muurolene	1493	n.d.	woody	n.d.	9	n.d.	n.d.	/	/	/	MS,RI,O
104	γ-Cadinene	1505	n.d.	herbal, woody	n.d.	n.d.	1	n.d.	/	/	/	MS,RI,O
105	α-Amorphene	1506	n.d.	woody, earthy	n.d.	n.d.	n.d.	1	/	/	/	MS,RI,O
106	β-Cadinene	1517	n.d.	green, woody	1	1	n.d.	n.d.	/	/	/	MS,RI,O
107	α-Calacorene	1536	n.d.	woody	n.d.	n.d.	n.d.	n.d.	/	/	/	MS,RI
108	1-Hydroxy-1,7-dimethyl-4-isopropyl-2,7-cyclodecadiene	1570	n.d.	woody, earthy, herbal	n.d.	n.d.	n.d.	n.d.	/	/	/	MS,RI

Table S3 (continued)

NO.	Name	RI[1]		Odor description	FD factors				Previously reported			identification
		HP-5	DB-WAX		S1	S2	S3	S4	A	B	C	
109	Humulene oxide II	1599	n.d.	woody, earthy	n.d.	n.d.	n.d.	n.d.	/	/	/	MS,RI
110	8-Heptadecene	1674	n.d.	oily, waxy, green	n.d.	n.d.	n.d.	n.d.	/	/	/	MS,RI
111	geranyl-α-terpinene	1984	n.d.	citrus, woody	n.d.	n.d.	n.d.	n.d.	/	/	√	MS,RI
112	Germacrene B	1548	n.d.	woody, earthy, spicy	n.d.	n.d.	n.d.	n.d.	/	/	/	MS,RI
113	Styrene	892	n.d.	sweet, balsamic, floral	n.d.	n.d.	n.d.	n.d.	/	/	/	MS,RI
**Others**												
114	p-Xylene	871	n.d.	sweet, aromatic	n.d.	n.d.	n.d.	n.d.	/	/	/	MS,RI,S
115	Bicyclo[5.1.0]octane, 8-(1-methylethylidene)-	1204	n.d.	woody, earthy	1	n.d.	n.d.	n.d.	/	/	/	MS,RI,O
116	Bicyclo[5.1.0]octane	1209	n.d.	woody, earthy, herbal	n.d.	n.d.	n.d.	n.d.	/	/	/	MS,RI
117	(-)-β-Elemene	1386	n.d.	sweet	n.d.	9	n.d.	n.d.	/	/	/	MS,RI,O
118	(E)-Sesquisabinene hydrate	1583	n.d.	woody, earthy, herbal	n.d.	n.d.	n.d.	n.d.	/	/	/	MS,RI
119	5-Methoxy-2,2,6-trimethyl-1-(3-methyl-buta-1,3-dienyl)-7-oxa-bicyclo[4.1.0]heptane	1724	n.d.	woody, herbal	n.d.	n.d.	n.d.	n.d.	/	/	/	MS,RI
120	Cembrene	1952	n.d.	woody	n.d.	n.d.	n.d.	n.d.	/	/	/	MS,RI
121	o-Cymene	1023	n.d.	citrus, herbal, spicy	n.d.	n.d.	n.d.	n.d.	/	/	/	MS,RI,S
**Unknow**												
122	Unknow1	1466	n.d.	n.d.	n.d.	n.d.	n.d.	n.d.	/	/	/	O
123	Unknow2	1544	n.d.	n.d.	n.d.	n.d.	n.d.	n.d.	/	/	/	O
124	Unknow3	1567	n.d.	n.d.	n.d.	n.d.	n.d.	n.d.	/	/	/	O
125	Unknow4	1645	n.d.	n.d.	n.d.	n.d.	n.d.	n.d.	/	/	/	O
126	Unknow5	1656	n.d.	n.d.	n.d.	n.d.	n.d.	n.d.	/	/	/	O
127	Unknow6	1748	n.d.	n.d.	n.d.	n.d.	n.d.	n.d.	/	/	/	O
128	Unkonwn7	1517	n.d.	woody	n.d.	n.d.	n.d.	n.d.	/	/	/	O

[a] Retention index (RI) on DB-WAX and HP-5 column were computed using n-alkanes (C7-C30) based on GC-MS-O analysis. [b] Previously reported: A [5], B [6] and C [7]. [c] Abbreviations: MS:

odor compounds were identified by NIST 11.L MS database; RI: odor compounds were identified by theoretical RI (https://webbook.nist.gov/chemistry/); O: olfactory determination; S: standard compounds determination. n.d.: not detected.

Table S4. GC-MS quantification of *Z. bungeanum* volatile fractions from solvent-extracted pericarp extracts (S1), steam-distilled pericarp essential oils (S2), steam-distilled leaf essential oils (S3), and solvent-extracted leaf extracts (S4).

NO.	Name	S1(μg/mg)	S2(μg/mg)	S3(μg/mg)	S4(μg/mg)
1	(+)-citronellal	n.d.	0.45±0.01[b]	0.46±0.01[a]	n.d.
2	Decanal	n.d.	n.d.	1.06±0.05[a]	n.d.
3	Cuminaldehyde	0.31±0.01[b]	0.35±0.02[a]	n.d.	0.06±0.01[c]
4	Phellandral	1.56±0.04[b]	2.22±0.03[a]	n.d.	n.d.
5	Undecanal	n.d.	n.d.	0.67±0.05[a]	n.d.
6	Z,Z-10,12-Hexadecadienal	n.d.	n.d.	1.22±0.02[a]	n.d.
7	Pentadecanal	n.d.	n.d.	0.34±0.01[a]	n.d.
8	3,5-Octadien-2-one	n.d.	n.d.	n.d.	0.09±0.01[a]
9	Carvone	0.52±0.03[b]	0.55±0.01[a]	n.d.	n.d.
10	2-Isopropyl-5-methyl-3-cyclohexen-1-one	n.d.	3.26±0.04[a]	n.d.	n.d.
11	Piperitone	n.d.	n.d.	0.44±0.01[a]	0.17±0.02[b]
12	Ionone	n.d.	n.d.	0.99±0.01[a]	n.d.
13	2-Undecanone	n.d.	n.d.	n.d.	0.11±0.01[a]
14	1-(3,6,6-Trimethyl-1,6,7,7a-tetrahydrocyclopenta[c]pyran-1-yl)ethanone	n.d.	n.d.	2.85±0.08[a]	n.d.
15	(E)-β-Ionone	n.d.	n.d.	n.d.	0.78±0.01[a]
16	Hexahydrofarnesyl acetone	n.d.	n.d.	0.45±0.01[a]	0.04±0.00[b]
17	(E,E)-Farnesyl acetone	n.d.	n.d.	0.21±0.01[a]	n.d.
18	cis-β-Terpineol	3.66±0.06[a]	1.66±0.02[b]	n.d.	0.06±0.00[c]
19	Linalool	10.86±0.45[b]	17.56±0.58[a]	9.65±0.32[c]	0.75±0.02[d]
20	trans-para-2-Menthen-1-ol	n.d.	2.01±0.03[a]	0.52±0.01[b]	n.d.
21	4-Carvomenthenol	2.02±0.06[c]	10.36±0.06[a]	2.60±0.15[b]	n.d.
22	α-Terpineol	5.45±0.46[b]	19.85±0.62[a]	3.96±0.09[c]	0.99±0.03[d]
23	γ-Terpineol	62.05±0.79[a]	n.d.	n.d.	0.23±0.01[b]
24	(Z)-Piperitol	n.d.	0.22±0.01[a]	n.d.	n.d.
25	Plinol C	n.d.	n.d.	0.56±0.03[a]	n.d.
26	5-Caranol, (1S,3R,5S,6R)-(-)-	0.41±0.03[a]	n.d.	n.d.	n.d.
27	4-epi-Cubebol	n.d.	n.d.	0.56±0.01[a]	n.d.
28	α-Elemol	n.d.	n.d.	n.d.	0.29±0.01[a]
29	Nerolidol	n.d.	0.78±0.02[b]	1.25±0.01[a]	0.12±0.01[c]
30	7-epi-cis-sesquisabinene hydrate	n.d.	0.37±0.01[a]	n.d.	n.d.
31	Cubenol	n.d.	0.45±0.01[b]	0.55±0.01[a]	n.d.
32	α-Acorenol	n.d.	0.44±0.01[a]	n.d.	n.d.
33	T-Cadinol	n.d.	3.11±0.06[b]	3.88±0.02[a]	0.12±0.01[c]
34	α-Cadinol	n.d.	3.25±0.05[a]	3.33±0.06[a]	0.11±0.03[b]
35	7-epi-trans-sesquisabinene hydrate	n.d.	0.89±0.02[a]	n.d.	n.d.
36	α-Bisabolol	n.d.	n.d.	n.d.	0.31±0.01[a]
37	5-Hydroxymethyl-1,1,4a-trimethyl-6-methylenedecahydronaphthalen-2-ol	n.d.	n.d.	n.d.	0.12±0.01[a]
38	Heptyl acetate	n.d.	n.d.	0.33±0.01[a]	n.d.
39	Octyl acetate	n.d.	n.d.	1.01±0.01[a]	n.d.
40	Linalyl acetate	120.82±1.06[a]	68.62±0.17[b]	41.47±1.02[c]	4.35±0.06[d]

Table S4 (continued)

NO.	Name	S1(μg/mg)	S2(μg/mg)	S3(μg/mg)	S4(μg/mg)
41	4-Terpinenyl acetate	3.56±0.08[b]	5.55±0.56[a]	1.66±0.06[c]	n.d.
42	Isopulegyl acetate	n.d.	2.21±0.03[b]	4.60±0.08[a]	0.23±0.01[c]
43	Myrtenyl acetate	n.d.	n.d.	1.12±0.01[a]	0.06±0.01[b]
44	2-Acetoxy-1,8-cineole	n.d.	1.79±0.02[a]	0.88±0.05[b]	0.33±0.02[c]
45	α-Terpinyl acetate	21.88±0.99[c]	26.37±0.53[b]	124.16±0.21[a]	4.35±0.26[d]
46	Citronellyl acetate	2.67±0.03[a]	2.25±0.03[b]	n.d.	n.d.
47	Geranyl acetate	n.d.	2.16±0.03[a]	2.16±0.01[a]	0.006±0.00[b]
48	Geranyl isovalerate	n.d.	n.d.	n.d.	0.07±0.01[a]
49	Butyl octyl phthalate	n.d.	n.d.	n.d.	0.02±0.01[a]
50	Methyl 11-hexadecenoate	n.d.	0.12±0.01[a]	n.d.	n.d.
51	Methyl palmitate	0.08±0.01[b]	0.12±0.01[a]	n.d.	n.d.
52	Dibutyl phthalate	0.01±0.00[a]	n.d.	n.d.	n.d.
53	Methyl 8-octadecenoate	n.d.	0.44±0.01[a]	n.d.	n.d.
54	Ethyl 9α-linolenate	n.d.	n.d.	0.55±0.01[a]	n.d.
55	Dihydroactinidiolide	n.d.	n.d.	n.d.	0.83±0.01[a]
56	Bornyl acetate	0.52±0.01[b]	2.34±0.02[a]	n.d.	0.04±0.00[c]
57	Carvyl acetate	n.d.	0.46±0.01[a]	n.d.	n.d.
58	Neryl acetate	0.23±0.01[b]	0.56±0.01[a]	0.54±0.06[a]	0.22±0.01[b]
59	Farnesyl acetate	n.d.	0.45±0.01[a]	n.d.	n.d.
60	2H-1-Benzopyran, 3,4,4a,5,6,8a-hexahydro-2,5,5,8a-tetramethyl-,	n.d.	n.d.	1.12±0.05[a]	n.d.
61	Manoyl oxide	n.d.	0.11±0.00[b]	0.22±0.01[a]	n.d.
62	Caryophyllene oxide	0.002±0.00[c]	0.002±0.00[b]	0.36±0.01[a]	0.001±0.00[c]
63	Ledene oxide	n.d.	n.d.	n.d.	0.26±0.01[a]
64	cis-Z-α-Bisabolene epoxide	n.d.	n.d.	n.d.	0.12±0.01[a]
65	Diepicedrene-1-oxide	n.d.	0.95±0.03[a]	n.d.	n.d.
66	Isoaromadendrene epoxide	n.d.	n.d.	1.09±0.01[a]	n.d.
67	Eucalyptol	n.d.	n.d.	49.75±0.05[a]	n.d.
68	α-Thujene	1.23±0.02[a]	0.6.2±0.01[b]	0.001±0.00[c]	0.002±0.00[c]
69	α-Pinene	2.02±0.01[c]	2.20±0.01[b]	5.72±0.12[a]	n.d.
70	Sabinene	7.72±0.03[a]	7.28±0.67[a]	7.82±0.44[a]	0.26±0.01[b]
71	β-Myrcene	2.41±0.04[b]	2.61±0.13[a]	1.51±0.03[c]	n.d.
72	β-Pinene	n.d.	n.d.	n.d.	0.76±0.09[a]
73	α-Phellandrene	n.d.	0.34±0.01[b]	0.68±0.06[a]	n.d.
74	α-Terpinene	0.006±0.00[c]	3.53±0.03[a]	1.72±0.01[b]	0.001±0.00[d]
75	Limonene	29.91±0.25[b]	31.56±0.88[a]	n.d.	0.005±0.00[c]
76	(E)-beta-Ocimene	2.85±0.01[c]	3.66±0.03[b]	6.46±0.04[a]	0.11±0.00[d]
77	.beta.-Ocimene	2.12±0.04[b]	3.64±0.04[c]	6.11±0.01[a]	0.35±0.01[d]
78	γ-Terpinene	0.96±0.03[c]	2.66±0.29[a]	1.65±0.09[b]	0.23±0.01[d]
79	Terpinolene	4.80±0.01[a]	1.50±0.02[b]	1.16±0.01[c]	0.001±0.00[d]
80	Alloocimene	n.d.	0.45±0.01[b]	0.77±0.06[a]	0.001±0.00[c]
81	para-Menthatriene	n.d.	n.d.	0.33±0.00[a]	n.d.
82	1,5,5-Trimethyl-6-methylene-cyclohexene	n.d.	n.d.	n.d.	0.04±0.00[a]

Table S4 (continued)

NO.	Name	S1(μg/mg)	S2(μg/mg)	S3(μg/mg)	S4(μg/mg)
83	Copaene	0.88±0.01[b]	0.79±0.02[c]	1.12±0.01[a]	0.12±0.01[d]
84	β-Bourbonene	n.d.	n.d.	0.06±0.01[a]	0.08±0.01[a]
85	α-Gurjunene	n.d.	n.d.	n.d.	0.05±0.01[a]
86	Caryophyllene	0.008±0.00[d]	0.006±0.00[c]	45.53±0.24[a]	1.21±0.04[b]
87	β-Cubebene	n.d.	n.d.	n.d.	0.36±0.01[a]
88	β-Copaene	n.d.	n.d.	1.36±0.01[a]	n.d.
89	γ-Elemene	0.65±0.02[b]	0.66±0.02[b]	1.31±0.06[a]	0.31±0.01[c]
90	β-Ylangene	n.d.	n.d.	n.d.	0.16±0.01[a]
91	Humulene	0.89±0.03[b]	0.88±0.01[b]	13.66±0.23[a]	0.65±0.05[c]
92	Alloaromadendrene	n.d.	0.55±0.01[b]	2.07±0.01[a]	0.44±0.01[c]
93	(+)-Calarene	n.d.	n.d.	1.22±0.03[a]	0.45±0.01[b]
94	Cedrene	n.d.	0.21±0.01[a]	n.d.	n.d.
95	α-Elemene	n.d.	n.d.	0.66±0.01[a]	n.d.
96	γ-Muurolene	0.77±0.02[b]	0.55±0.01[c]	1.66±0.01[a]	0.11±0.01[d]
97	Germacrene D	2.35±0.03[b]	1.82±0.03[b]	26.66±0.81[a]	0.67±0.01[c]
98	Valencene	n.d.	n.d.	1.43±0.01[a]	0.11±0.00[b]
99	β-Selinene	n.d.	0.67±0.02[a]	n.d.	n.d.
100	Bicyclosesquiphellandrene	n.d.	n.d.	1.56±0.01[a]	n.d.
101	α-Selinene	0.98±0.01[a]	0.88±0.01[b]	n.d.	n.d.
102	Eremophilene	n.d.	n.d.	4.68±0.09[a]	0.34±0.01[b]
103	α-Muurolene	1.65±0.03[b]	1.35±0.02[c]	5.46±0.01[a]	n.d.
104	γ-Cadinene	n.d.	n.d.	8.20±0.10[a]	n.d.
105	α-Amorphene	3.23±0.06[a]	2.60±0.04[b]	n.d.	0.34±0.01[c]
106	β-Cadinene	3.56±0.07[c]	4.15±0.07[b]	14.65±0.04[a]	0.65±0.01[d]
107	α-Calacorene	n.d.	0.22±0.01[b]	1.16±0.01[a]	n.d.
108	1-Hydroxy-1,7-dimethyl-4-isopropyl-2,7-cyclodecadiene	1.12±0.01[a]	n.d.	n.d.	n.d.
109	Humulene oxide II	n.d.	n.d.	n.d.	0.22±0.01[a]
110	8-Heptadecene	0.45±0.01[b]	0.78±0.01[a]	n.d.	n.d.
111	geranyl-α-terpinene	n.d.	0.27±0.01[a]	n.d.	n.d.
112	Germacrene B	2.06±0.02[c]	3.20±0.05[b]	4.60±0.01[a]	n.d.
113	Styrene	n.d.	n.d.	n.d.	0.12±0.01[a]
114	p-Xylene	n.d.	n.d.	n.d.	0.006±0.00[a]
115	Bicyclo[5.1.0]octane, 8-(1-methylethylidene)-	0.34±0.01[a]	n.d.	n.d.	n.d.
116	Bicyclo[5.1.0]octane	n.d.	n.d.	0.45±0.01[a]	n.d.
117	(-)-β-Elemene	n.d.	1.75±0.05[b]	9.65±0.02[a]	0.87±0.01[c]
118	(E)-Sesquisabinene hydrate	n.d.	0.450±0.01[a]	n.d.	n.d.
119	5-Methoxy-2,2,6-trimethyl-1-(3-methyl-buta-1,3-dienyl)-7-oxa-bicyclo[4.1.0]heptane	n.d.	n.d.	0.66±0.01[a]	n.d.
120	Cembrene	n.d.	0.88±0.01[a]	n.d.	n.d.
121	o-Cymene	0.07±0.01[b]	0.15±0.01[a]	0.02±0.00[c]	n.d.
122	Unknow1	n.d.	n.d.	n.d.	0.08±0.01[a]
123	Unknow2	n.d.	n.d.	n.d.	0.16±0.01[a]

TableS4(continued)

NO.	Name	S1(μg/mg)	S2(ug/mg)	S3(ug/mg)	S4(ug/mg)
124	Unknow3	n.d.	n.d.	n.d.	1.13±0.01[a]
125	Unknow4	n.d.	n.d.	n.d.	1.1±0.03[a]
126	Unknow5	n.d.	n.d.	n.d.	0.51±0.01[a]
127	Unknow6	n.d.	n.d.	n.d.	0.81±0.35[a]
128	Unkonwn7	n.d.	n.d.	n.d.	0.61±0.03[a]

There is no significant difference when the mark contains the same letter (a, b, c, d ($p < 0.05$)). n.d.: not detected.

Table S5. HS-GC-IMS identification of *Z. bungeanum* volatile fractions from solvent-extracted pericarp extracts (S1), steam-distilled pericarp essential oils (S2), steam-distilled leaf essential oils (S3), and solvent-extracted leaf extracts (S4).

No.	Compound[a]	Odor describes	RI[b]	Rt [sec][c]	Dt [RIPrel][d]	Comment
**Aldehydes**						
A1	2-Methylpropanal	floral，green	593.1	81.394	1.0863	
A2	2-Methyl-2-propenal	hyacinth，foliage，green	572.5	71.314	1.228	
A3	Butanal(M)	pungent, diffusive, cocoa, choking	577.8	73.889	1.1207	Monomer
A4	Butanal(D)	pungent, diffusive, cocoa, choking	584.5	77.179	1.2692	Dimer
A5	2-Methyl-2-pentenal	pungent, fruity，green	843.5	290.342	1.1633	
A6	4-Methylbenzaldehyde	fruity	1071.6	641.316	1.1915	
A7	2-Methylpentanal	etherial, green，vegetative fruity nuance	750.5	204.408	1.2202	
A8	Hexanal	green, woody	809.7	262.357	1.2546	
A9	(-)-Perillaldehyde	green，oily，fatty，minty	1147.1	776.585	1.2961	
A10	Anisaldehyde	floral,herbaceous，woody	1326.3	1097.801	1.6676	
A11	2,4-Heptadienal	green，pungent，fruity，spicy	1000.2	513.326	1.1916	
**Alcohols**						
A12	2-Hexanol	winey，fruity，fatty，terpenic	813.4	265.401	1.2886	
A13	(Z)-3-octen-1-ol	fatty，fruity，herbal，spicy	1065.2	629.767	1.2947	
A14	2-Methyl-1-pentanol	pungent, fermented fruity	831.2	280.113	1.5769	
A15	2-Octanol	fresh，spicy，green，woody，herbal	1014.1	538.119	1.4319	
A16	1-Pentanol	pungent, fermented,	763.1	217.92	1.2548	
A17	2-Heptanol(M)	lemongrass，herbal，sweet，floral	882	327.027	1.3666	Monomer
A18	2-Heptanol(D)	citrus，herbal，floral	1325.5	1096.286	1.7086	Dimer
A19	2-Methoxy-4-vinylphenol	woody，fresh	1325.4	1096.218	1.747	
A20	α-Terpineol	terpenic, citrus, woody, floral	1173.3	823.476	1.2209	
A21	4-Carvomenthenol	woody, mentholic, citrus, terpenic, spicy	1163.2	805.487	1.7286	
**Ketones**						
A22	2,3-Butanedione	sweet, creamy, buttery, pungent	599.3	84.433	1.1781	
A23	3-Octanone	fresh，herbal，lavender	999.6	512.214	1.2926	
A24	Isophorone	cooling, woody, sweet, green	1133.5	752.243	1.2514	
A25	3-Hepten-2-one(M)	green,grassy	922.6	380.507	1.2165	Monomer
A26	3-Hepten-2-one(D)	green,grassy	946.5	418.901	1.2221	Dimer
A27	3-Methyl-2-pentanone	fresh，herbal，fruity	737.3	189.995	1.1773	
A28	Cyclopentanone	minty	801.8	255.583	1.1155	
A29	6-Methylhepta-3,5-dien-2-one	cinnamon，coconut，spice，woody	1105.3	701.662	1.723	
**Acids**						
A30	Pentanoic acid	acidic and sharp	905.5	356.066	1.2171	
A31	Hexanoic acid	mild, fatty	1018.6	546.22	1.2899	
A32	2-Methylpropanoic acid	acidic，sour，rancid	772.9	228.179	1.6639	
**Esters**						
A33	Methyl acetate	etherial and solvent-like	838.7	286.319	1.0437	
A34	Methyl hexanoate	ethereal，fruity，pineapple	947.4	420.325	1.2896	
A35	2-Butoxyethyl acetate	ethereal，fruity	902.2	351.544	1.2928	

TableS5(continued)

No.	Compound[a]	Odor describes	RI[b]	Rt [sec][c]	Dt [RIPrel][d]	Comment
A36	Isopropyl 2-methylbutanoate	fruity, sweet, green, oily	857	302.155	1.2919	
A37	Ethyl 3-hydroxybutanoate	fruity，green	955.3	433.801	1.6373	
A38	Methyl heptanoate	sweet, fruity and green	1013.4	536.867	1.7924	
A39	Neryl acetate	floral, citrus	1328.5	1101.766	1.2269	
A40	Amyl acetate	ethereal，fruity	1209.5	888.448	1.2974	
A41	Isoamyl acetate(M)	sweet, banana, fruity	879.4	324.182	1.309	Monomer
A42	Isoamyl acetate(D)	sweet, banana, fruity	882.9	328.079	1.7484	Dimer
A43	Acetic acid, 2-methylpropyl ester(M)	estery, fruity	793.8	248.452	1.2171	Monomer
A44	Acetic acid, 2-methylpropyl ester(D)	estery, fruity	786.8	241.886	1.6158	Dimer
A45	Isopentyl formate	sharp, green	775.6	230.9	1.2897	
A46	Ethyl heptanoate	fruity, sweet, green	1323.9	1093.412	1.9308	
A47	Ethyl butanoate	ethereal, fruity	806.7	259.778	1.5792	
A48	Hexyl isobutyrate	sweet, green, fruity	1326	1097.284	1.4508	
**Heterocyclic compounds**						
A49	Sotolone	sweet, caramellic, maple	1122	731.593	1.2237	
A50	5-Methylquinoxaline	nutty，musty	1537.4	1476.094	1.2232	
A51	2-Isobutyl-3-Methoxypyrazine	green，pepper	1191.7	856.535	1.8243	
A52	4,5-Dihydro-3(2H)-thiophenone	garlic，meaty，green	947.4	420.263	1.4277	
A53	γ-Nonalactone	coconut，creamy，waxy	1325.2	1095.852	1.9054	
A54	2-Butanoyl furan	balsamic	1105.1	701.222	1.6593	
A55	2-Acetyl-1-pyrroline	popcorn，toasted，grain，malty	934.7	399.527	1.1267	
**Alkene (only Monoterpenes)**						
A56	β-Myrcene(M)	terpenic, herbal, woody	976.9	471.625	1.2221	Monomer
A57	β-Myrcene(D)	terpenic, herbal, woody	973.8	466.103	1.6416	Dimer
A58	β-Pinene	cooling, woody, piney	999.1	511.251	1.2163	
A59	Δ-3-Carene	citrus，terpenic，herbal，woody	1014.6	539.132	1.2112	
A60	β-Ocimene	citrus, green, terpenic, woody	1040.8	586.085	1.2508	
A61	α-Pinene(M)	woody, terpenic, herbal	924.7	383.832	1.6608	Monomer
A62	α-Pinene(D)	woody, terpenic, herbal	922.7	380.702	1.695	Dimer
A63	α-Pinene(T)	woody, terpenic, herbal	925	384.279	1.7364	Trimers
A64	Limonene	citrus	1024.4	556.591	1.7322	
A65	p-Cymene	fresh，citrus，terpenic，woody，spicy	1005.8	523.384	1.7288	
A66	Camphene	woody，herbal	954.7	432.753	1.7368	
A67	α-Terpinene	citrus, woody, terpenic	984.2	484.658	1.7248	
A68	α-Phellandrene	citrus, herbal, terpenic, green	1002.2	516.919	1.6718	
**Other compounds**						
A69	Dimethylamine	fishy	583.9	76.885	1.0665	
A70	Methanethiol	vegetable oil, alliaceous	659.7	117.934	1.0376	
A71	(E)-3-Pentenenitrile	pungent，musty	692.3	143.726	1.1565	

TableS5(continued)

No.	Compound[a]	Odor describes	RI[b]	Rt [sec][c]	Dt [RIPrel][d]	Comment
A72	1-Propanethiol	cabbage，gassy，sweet，onion	618.9	94.128	1.1613	
**Unkonwn**						
A73	Compound from HS-Vial Septum	/	831.1	279.978	1.4614	

[a]Aroma compounds were identified based on standards, NIST 11, and IMS database. [b]Retention index calculated on MXT-5 column using N-ketones (C4-C9). [c]Retention time in the capillary GC column. [d]Drift time in the drift tube.

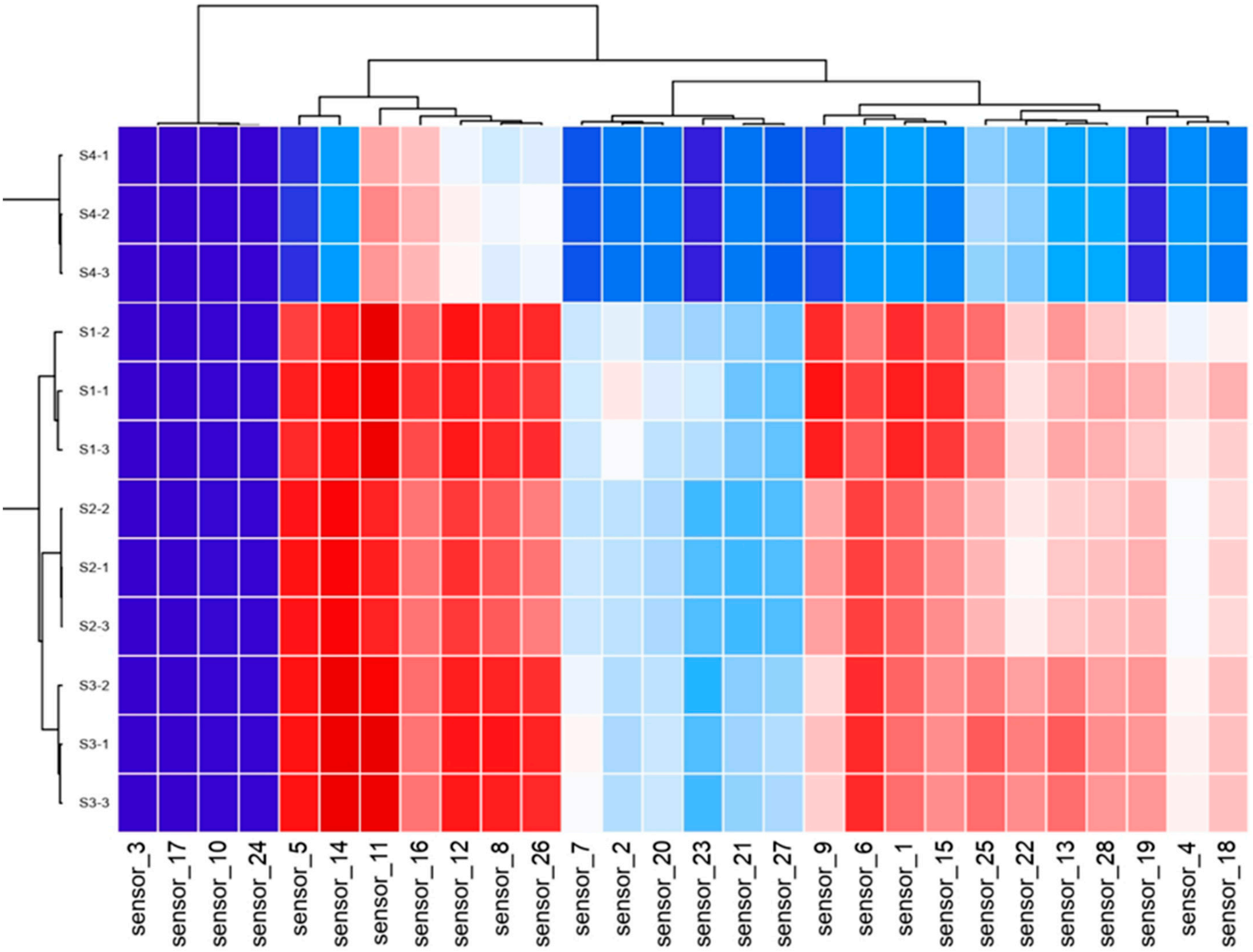

Figure S1. Cluster heat map of *Z. bungeanum* volatile fractions from S1-S4 via E-nose.

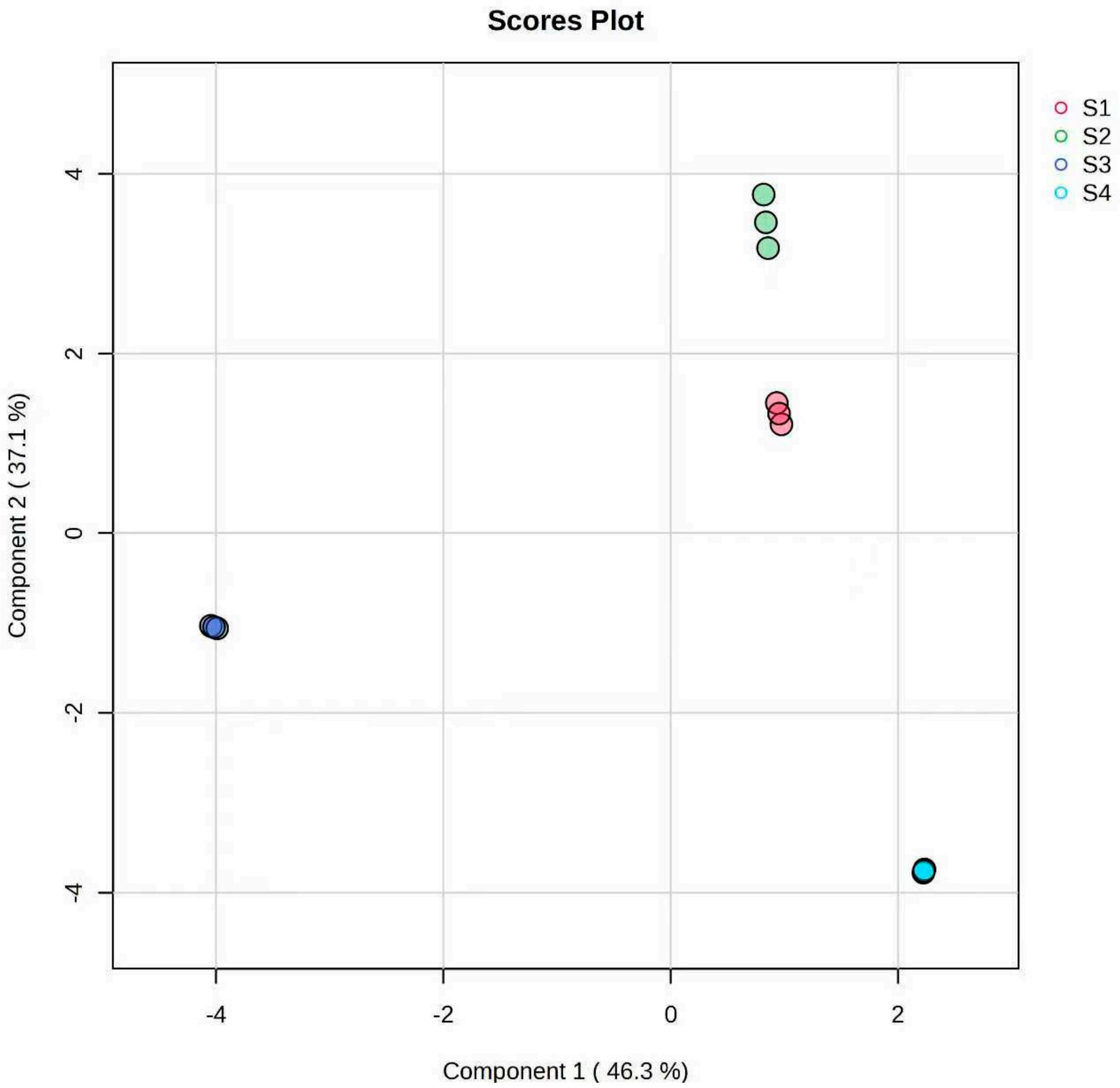

Figure S2. sPLS-DA analysis of *Z. bungeanum* volatile fractions from S1-S4.

Supplementary Materials and Methods

2.1 Materials

Fresh leaves and pericarps of Z. bungeanum (Dwarf thornless "Dahongpao" Chinese prickly ash) were obtained from Hubei Mingchuang Agricultural Technology Devel-opment Co., Ltd. (Hong'an, Hubei, China). The plant materials were harvested during the mid-August 2023 harvest season. The pericarps were collected at the commercial maturity stage, defined visually by a fully developed red coloration and the initial de-hiscence of the follicle. The leaves were harvested concurrently from the same trees to ensure a rigorous matrix-matched comparison.

The geographic coordinates of the cultivation site are approximately 31°16'N, 114°37. Upon collection, fresh leaves were immediately transported to the laboratory under cold chain conditions and either used immediately or stored at -20 °C. The peri-carps were dried at 60 °C and similarly stored at -20 °C prior to volatile extraction.

2.3. Sensory evaluation

The sensory evaluation was performed referring to prior sensory reports with slight modification[5]. To ensure the reliability and reproducibility of the sensory data, the selection and training of the sensory panel were conducted in accordance with international standards (ISO 8586).

Initial candidates were recruited from the student and staff population at Wuhan Polytechnic University. The preliminary selection criteria required candidates to be non-smokers, have no known allergies to spices or essential oils, and possess normal olfactory and gustatory functions. 25 candidates underwent a preliminary screening test to assess their basic sensory acuity, which included odor recognition tests using common aroma standards and an intensity ranking test. Based on their performance, sensitivity, and availability, 15 candidates (7 males and 8 females, aged 22-32 years old) were selected to form the analytical sensory panel.

The selected panelists underwent a rigorous training program consisting of 6 sessions, with each session lasting approximately 1 hour, held over three weeks. The training was structured as follows: (1) Vocabulary Generation and Familiarization: Panelists were introduced to the odor attributes (citrus, floral, herbal, spicy, woody, and minty) and were trained to identify them using the specific chemical reference standards detailed in Table A1. (2) Intensity Scaling: Panelists were trained to utilize a 6-point intensity scale ranging from 0 (not perceivable) to 5 (strongly perceivable). To standardize perception, varying concentrations of the reference standards were presented, and the panel iteratively discussed the perceived intensities until a consensus was reached on how to anchor the scale. (3) Simulated Matrix Evaluation: In the final training phases, panelists evaluated mock samples that simulated the complex matrix of *Z. bungeanum* fractions.

At the conclusion of the training, all evaluators received and passed a final perception and reproducibility test to participate in the experiment. For the formal Aroma Profiling Analysis (APA), all sessions were conducted in a dedicated sensory evaluation room at 24±1 °C. Samples (10 μL) were placed into 15 mL glass vials, allowed to equilibrate, and then evaluated for overall odor characteristics blindly in randomized order. The sealed vials were allowed to equilibrate at 24±1 °C for 30 minutes prior to evaluation. During the sessions, samples were coded with 3-digit random numbers and presented blindly to the panelists in a balanced, randomized order. To control for interfering factors and prevent olfactory fatigue, panelists were required to take a 3-minute break between each sample evaluation and were provided with fresh ambient air to neutralize their olfactory receptors. Panelists unsealed the vials solely at the moment of evaluation to assess the overall odor characteristics.

2.4. Electronic nose (E-nose) analysis

The volatile fingerprints of S1-S4 were distinguished using a cNose-18 system (Shanghai Baosheng Industrial Development Co., Ltd.) equipped with 28 metal oxide sensors. Following the modified metho [6], 1.0 μL of the sample was placed in a 10 mL headspace vial, which was immediately sealed with a PTFE/silicone septum. To ensure the generation of steady headspace volatiles, the sealed vials were equilibrated in a dry bath at 40 °C for 15 min prior to extraction.

For the system operating conditions, clean, dry air (purity ≥ 99.99%) was utilized as the carrier gas at a constant flow rate of 1.0 L/min. Prior to the first sample, the sensor array was stabilized with a warm-up phase of 10 min. The measurement duration for each sample was set to 120 s to allow the sensor signals to reach a steady state, followed by a purging/cleaning phase of 180 s using the carrier gas to return the sensors to their baseline response.

During data processing, the maximum response values of each sensor were ex-tracted by calculating the ratio of the maximum conductance of the sensor in the sam-ple gas (G) to its baseline conductance in the clean carrier gas (G0). Each sample was tested in triplicate.

2.5. HS-GC-IMS analysis

Volatile profiling was performed using an HS-GC-IMS system (FlavourSpec®, G.A.S., Germany) according to Wu et al.[7] with modifications. For sample preparation, 0.1 μL of the pure sample was placed into a 20 mL headspace vial, which was immedi-ately sealed with a magnetic crimp cap equipped with a silicone septum. The sealed vials were incubated at 35 °C for 10 min under constant agitation (500 rpm) to achieve headspace equilibrium prior to analysis.

The temperature and volume of the headspace injection were 40 °C and 100 μL, respectively. Due to the instrument's hardware configuration, the sample was intro-duced in a strictly splitless (direct injection) mode. Ultrapure nitrogen (purity ≥ 99.999%) was used as a carrier gas and the initial flow rate set to 1 mL/min, raised to 2 mL/min within 3 min, then raised to 10 mL/min within 5 min, then raised to 12 mL/min within 7 min, then raised to 100 mL/min within 20 min, and finally raised to 150 mL/min within 30 min. The drift gas flow rate was established at 150 mL/min. The MXT-5 column (15 m × 0.53 mm, 1 μm, Restek Corporation, USA) and IMS temperature were kept at 60 °C and 45 °C. The IMS detector operated in positive ion mode with a drift tube voltage of 5000 V. The data were qualitatively analyzed using VOCal software (version 0.4.03), which is equipped with NIST 11 and IMS databases.

2.6. Isolation of aroma compounds by SPME

For GC-MS-O analysis, volatiles were extracted using headspace solid-phase mi-croextraction (HS-SPME)[8]. Prior to initial use, the 50/30 μm CAR/DVB/PDMS fiber (Supelco, Inc., Bellefonte, USA) was conditioned in the GC injection port at 270 °C for 30 min according to the manufacturer's recommendations.

For sample preparation, 1 μL of the volatile fraction was placed into a 20 mL glass headspace vial. The vials were immediately sealed with silicone septa and aluminum crimp caps. To ensure complete volatilization and partition equilibrium in the headspace, the sealed vials were equilibrated at 80 °C for 15 min under continuous agitation at 250 rpm. Following equilibration, the conditioned fiber was exposed to the sample head-space at 80 °C for 20 min.

Subsequently, the fiber was withdrawn and inserted into the GC injector for ther-mal desorption at 270 °C for 10 min. Due to the high volatile concentration of the frac-tions, the injection was performed in a split mode with a split ratio of 75:1, as further detailed in Section 2.7. To rigorously monitor and prevent any cross-contamination or fiber carryover, blank runs utilizing empty sealed vials were systematically performed between different sample extractions. Three parallel tests were performed for each sample.

2.7. Identification of the odor compounds by GC-MS-O

Evaluation of highly volatile odor compounds was accomplished by means of Ag-ilent 7890A-5975C GC-MS (Agilent Technologies Inc., USA) in combination with an ol-factometer (ODP3, Gerstel, Germany)[9]. To prevent condensation and mucosal dry-ing, the sniffing port was maintained at 250 °C and supplied with humidified air.

The SPME extract was injected into HP-5 (30 m×250 μm×0.25 μm). The GC analysis was performed as followed: split ratio, 75:1; injection temperature, 270°C; oven tem-perature program, 40 °C and hold for 1 min, up to 170 °C at 3 °C/min, up to 270 °C at 8 °C /min and hold for 5 min; the flow rate of carrier gas (high purity helium), 1.3 mL/min. MS conditions: ion source temperature, 250 °C; quadrupole temperature of 150 °C; EI energy, 70 eV; scan range, m/z 35-550; solvent delay time, 3.0 min.

Odor compounds with odor characteristics were recorded by at least three experi-enced team members (two females and one male, 24-32 years old). These assessors were selected from the trained panel described in Section 2.3. Odor events were synchro-nized with chromatographic peaks using the Gerstel ODP recording software. To en-sure reliability, odor descriptors were consolidated and considered valid only if inde-pendently perceived and agreed upon by at least two of the three assessors.

The data were qualitatively analyzed using MSD Chemstation (version F.01, Ag-ilent, USA), which is equipped with NIST11.L databases. Compound identification was confirmed based on three strict criteria: (1) a mass spectral match factor of ≥800; (2) confirmation of experimental retention indices (RIs) compared with literature values,

calculated using a homologous series of N-alkanes (C7-C40) analyzed under identical conditions; and (3) consensus of the perceived odor descriptors.

2.10. Analysis of odor activity values (OAVs)

Odor thresholds (OTs) were determined in a refined, odorless camellia oil matrix using the 3-alternative forced choice (3-AFC) method [10]. The evaluation panel con-sisted of the 15 trained assessors previously described in Section 2.3. Assessors were thoroughly familiarized with the target odorants prior to formal testing.

For the 3-AFC procedure, a serial dilution of each authentic standard was pre-pared in the odorless camellia oil using a dilution factor of 3. During the test, panelists were presented with three coded samples at each concentration level: two blanks (pure camellia oil) and one spiked sample. Panelists were required to identify the odd sample containing the odorant, progressing sequentially from the lowest to the highest con-centration. OTs determinations were conducted in a controlled sensory evaluation room at 25±1 °C.

The threshold determination protocol was strictly defined: an individual assessor's odor threshold was calculated as the geometric mean of the highest concentration they failed to recognize and the adjacent lowest concentration they correctly identified. The final group odor threshold for each compound was then aggregated by calculating the geometric mean of the individual thresholds from all 15 assessors. The odor activity value (OAV), which indicates a compound's contribution to the overall aroma, was calculated as the ratio of its concentration (C) to its OT.

2.11. Recombination and omission experiments

For aroma recombination, according to previous research, aroma compounds with OAVs ≥ 1 in each sample were added to odorless refined camellia oil based on their quantitative results to prepare the aroma recombination sample [11]. The respective recombinates were evaluated by the sensory panel as described above for APA. For omission experiments, triangle tests were carried out with one omitted sample and two recombinant samples according to previous research [6].

The omission tests were evaluated by twelve trained panelists (six males and six females) selected from the main sensory panel. To ensure robust statistical power, the evaluations were conducted in triplicate, yielding a total of 36 judgments per omission model. During the tests, all samples were labeled with 3-digit random codes and pre-sented blindly to the panelists in a balanced, randomized order to prevent any posi-tional or psychological bias. The statistical significance of each omission was deter-mined by counting the number of accurate identifications of the omitted sample. Sig-nificance ($p < 0.05$, $p < 0.01$, or $p < 0.001$) was established by comparing the correct re-sponses against the critical values derived from the binomial distribution for triangle tests, strictly adhering to ISO 4120 guidelines.

2.12. Statistical analysis

All instrumental analyses (E-nose, HS-GC-IMS, and GC-MS-O) were performed in triplicate for each sample. For the sensory evaluation, assessments were conducted in three independent repetitions by the 15 trained panelists, meaning that 45 individual observations formed the basis of the mean values calculated for each sensory attribute. The final results were expressed as mean ± standard deviation.

Significance analysis of the data ($p < 0.05$) was carried out using SPSS 22. Cluster heat maps were drawn using TBtools. Histograms, partial least-squares regression (PLSR), and sparse partial least squares discriminant analysis (sPLS-DA) were drawn using Origin 2021, UnscramblerX 10.4, and MetaboAnalyst 6.0, respectively.

Reference:

1. Wang, J.; Yang, F.; Guo, J.; Zou, T.; Liu, Y.; Song, H. Characterization of key aroma-active compounds in Zanthoxylum schinifolium by sensory evaluation and multiple instrumental analyses. *Journal of Food Composition Analysis* 2024, *136*, 106848, doi:10.1016/j.jfca.2024.106848.
2. Xiao, Z.; Li, Q.; Niu, Y.; Zhou, X.; Liu, J.; Xu, Y.; Xu, Z. Odor-active compounds of different lavender essential oils and their correlation with sensory attributes. *Industrial Crops and Products* 2017, *108*, 748-755, doi:10.1016/j.indcrop.2017.07.040.
3. Zarzo, M.J.C.P. Multivariate analysis and classification of 146 odor character descriptors. 2021, *14*, 79-101, doi:10.1007/s12078-021-09288-1.
4. Zhang, J.; Zhang, M.; Bhandari, B.; Wang, M. Basic sensory properties of essential oils from aromatic plants and their applications: A critical review. *Critical Reviews in Food Science and Nutrition* 2024, *64*, 6990-7003, doi:10.1080/10408398.2023.2177611.
5. Yin, W.T.; Ma, X.T.; Li, S.J.; Wang, X.D.; Liu, H.M.; Shi, R. Comparison of key aroma-active compounds between roasted and cold-pressed sesame oils. *Food Res Int* 2021, *150*, 110794, doi:10.1016/j.foodres.2021.110794.
6. Jia, X.; Zhou, Q.; Huang, D.; Zhang, N.; Qu, S.; An, Q.; Wang, Q.; Ren, J.; Zhang, H.; Pan, S.; et al. Insight into the comparison of key aroma-active compounds between camellia oils from different processing technology. *Food Chem* 2024, *430*, 137090, doi:10.1016/j.foodchem.2023.137090.
7. Wu, X.; Yin, J.; Ding, H.; Li, W.; Han, L.; Yang, W.; Li, F.; Song, X.; Bie, S.; Gong, X.J.F. The discrimination and characterization of volatile organic compounds in different areas of Zanthoxylum bungeanum pericarps and leaves by HS-GC-IMS and HS-SPME-GC-MS. 2022, *11*, 3745, doi:10.3390/foods11223745.
8. Cong, Y.; Lei, Y.; Xiao, Y.; Zhou, Q.; Wu, Z.; Feng, J.; Yang, T.; Zhang, W. Lipidomics and Flavouromics assessment of the effects of enzyme modification on butter composition. *Food Chem* 2025, *470*, 142655, doi:10.1016/j.foodchem.2024.142655.
9. Jia, X.; Yu, P.; An, Q.; Ren, J.; Fan, G.; Wei, Z.; Li, X.; Pan, S. Identification of glucosinolates and volatile odor compounds in microwaved radish (Raphanus sativus L.) seeds and the corresponding oils by UPLC-IMS-QTOF-MS and GC x GC-qMS analysis. *Food Res Int* 2023, *169*, 112873, doi:10.1016/j.foodres.2023.112873.
10. Al-Dalali, S.; Zheng, F.; Sun, B.; Chen, F. Characterization and Comparison of Aroma Profiles and Aroma-Active Compounds between Traditional and Modern Sichuan Vinegars by Molecular Sensory Science. *J Agric Food Chem* 2020, *68*, 5154-5167, doi:10.1021/acs.jafc.0c00470.
11. Neugebauer, A.; Schieberle, P.; Granvogl, M. Characterization of the Key Odorants Causing the Musty and Fusty/Muddy Sediment Off-Flavors in Olive Oils. *J Agric Food Chem* 2021, *69*, 14878-14892, doi:10.1021/acs.jafc.1c02228.
